# Pandemic 2009 H1N1 Influenza Venus reporter virus reveals broad diversity of MHC class II-positive antigen-bearing cells following infection in vivo

**DOI:** 10.1038/s41598-017-11313-x

**Published:** 2017-09-07

**Authors:** Anthony DiPiazza, Aitor Nogales, Nicholas Poulton, Patrick C. Wilson, Luis Martínez-Sobrido, Andrea J. Sant

**Affiliations:** 1 Department of Microbiology and Immunology, David H. Smith Center for Vaccine Biology and Immunology, University of Rochester Medical Center, Rochester, NY, 14642 USA; 2 Department of Medicine, Section of Rheumatology, The Committee on Immunology, The Knapp Center for Lupus and Immunology Research, The University of Chicago, Chicago, IL 60637 USA

## Abstract

Although it is well established that Influenza A virus infection is initiated in the respiratory tract, the sequence of events and the cell types that become infected or access viral antigens remains incompletely understood. In this report, we used a novel Influenza A/California/04/09 (H1N1) reporter virus that stably expresses the Venus fluorescent protein to identify antigen-bearing cells over time in a mouse model of infection using flow cytometry. These studies revealed that many hematopoietic cells, including subsets of monocytes, macrophages, dendritic cells, neutrophils and eosinophils acquire influenza antigen in the lungs early post-infection. Surface staining of the viral HA revealed that most cell populations become infected, most prominently CD45^neg^ cells, alveolar macrophages and neutrophils. Finally, differences in infection status, cell lineage and MHC class II expression by antigen-bearing cells correlated with differences in their ability to re-stimulate influenza-specific CD4 T cells *ex vivo*. Collectively, these studies have revealed the cellular heterogeneity and complexity of antigen-bearing cells within the lung and their potential as targets of antigen recognition by CD4 T cells.

## Introduction

Influenza A virus (IAV) infections cause seasonal epidemics as well as occasional pandemics and remain a major cause of morbidity and mortality worldwide^1, 2^. During the 2009 season, zoonotic transmission of a novel, swine-origin IAV emerged in the human population, causing a pandemic outbreak affecting more than 214 countries, resulting in at least 100,000 respiratory deaths within the first year of circulation^3–6^. For this reason, understanding influenza virus biology and immunity are critical for the development of effective countermeasures, including antiviral drugs and vaccines. Although it is well established that influenza infection is initiated in the respiratory tract, the sequence of events and the cell types that become infected or access viral antigens remains incompletely understood.

The lung is highly heterogeneous in cell composition, which can be divided into two major categories- bone marrow-derived and non-bone marrow-derived, on the basis of CD45 expression. CD45^neg^ cells are comprised mainly of epithelial, endothelial and stromal cells, whereas CD45^pos^ cells consist of subpopulations of lymphocytes and myeloid cells, including monocytes, macrophages (MΦ), dendritic cells (DC) and neutrophils (reviewed in refs 7–10). During the course of influenza infection, encounter of pulmonary cells with virus or viral antigens can lead to profound alterations in their fate and function, with immediate and downstream consequences on host immunity (reviewed in refs 10–13). For example, innate immune sensing of influenza triggers rapid release of interferons (IFNs) that control virus replication and dissemination by inducing an anti-viral state in nearby cells, thus making them resistant to viral infection^14^. Furthermore, IFNs and other pro-inflammatory mediators can lead to maturation of existing cells as well as recruitment of innate effectors from circulation^15, 16^. Finally, cells with the potential to transport antigen to the local draining lymph node(s) (dLN) can mediate pathogen clearance by initiating the adaptive immune response (reviewed in refs 8, 9 and 17).

Although cellular tropism, infectivity and antigen presentation has largely been studied *in vitro* (reviewed in refs 9 and 18), there is a need to identify the cells *in vivo* that become infected and access antigen over time within naturally occurring microenvironments elicited by influenza infection. *In vivo* studies are necessary because the cellular composition, activation state and cytokine milieu change dramatically in the respiratory tract following influenza infection^19–21^. An accumulation of evidence supports the concept that different cells access antigen by different pathways, either endogenously through infection or exogenously via uptake (reviewed in refs 22 and 23). While *in vivo* studies are limited, the current paradigm is that epithelial cells lining the upper and lower airways serve as the major target cells of influenza and are productively infected, leading to cell death and release of progeny virions^24–27^. Alveolar MΦ (aMΦ), CD103 DC and neutrophils are also among the first to encounter influenza and may also become infected *in vivo* through recognition by attachment factors (terminal sialic acid) and/or entry receptors (C-type lectin receptors) (reviewed in refs 28 and 29). While it is generally thought that infected aMΦ and neutrophils remain within the lung, CD103 DC can become protected from infection and have been shown to transport viral antigen to the local dLN and play a role in driving T-cell mediated immunity^30, 31^. Collectively, these studies have highlighted the importance of different antigen-bearing cells in host immunity. Responses are also likely to depend on the strain of influenza virus and host genetic background^32^.

Because many distinct cell types in the respiratory tract express an overlapping set of cell surface markers, resolution of individual populations at the single cell level has relied on the use of multi-parameter flow cytometry^33–37^. For example, although CD11c (αX integrin), CD11b (αM integrin) and Major Histocompatibility Complex (MHC) class II have historically been used to distinguish DCs from MΦ in the lung, emerging information from studies focused on defining cell type-specific markers have proven that this strategy is not sufficient^33–35^. Importantly however, recent and extensive studies by several groups have provided insights for the construction of polychromatic flow cytometry panels in combination with gating strategies that can identify the key cell types in the respiratory tract, both at steady-state and in the context of inflammation^33, 34, 36, 38, 39^. Accurate identification of distinct cell types is critical to better understand their importance in protective immunity. Additionally, despite advances in dissecting the cellular complexity that exists within the inflamed lung, very little is known about the identity, distribution and abundance of cells that access influenza antigens following infection.

One of the recent and powerful tools that has been used to identify cells that have encountered influenza are reporter-expressing viruses that are engineered to express bioluminescent or fluorescent proteins^25, 40–45^ upon infection. However, many reporter viruses constructed to date have suffered fitness costs including attenuated viral replication and/or loss of reporter activity^24, 25, 30, 43, 46–58^, making them unsuitable for studying the role and immunological consequences of antigen-bearing cells during the course of infection *in vivo*. Although efforts have been made to circumvent issues of viral replication and fluorescence stability through serial viral passaging in mice^58^, important virulence factors could be modified during this adaptation process. Furthermore, many of the previous studies have relied on the use of laboratory influenza strains (e.g. A/PR/8/34 (H1N1))^24, 30, 56, 59^. Accumulated studies have shown that the commonly used, mouse-adapted PR8 strain (Mt. Sinai) is atypical because it completely lacks glycosylation sites on the globular head of the viral hemagglutinin (HA) protein, which may affect viral tropism or interactions with carbohydrate receptors expressed by multiple innate immune cells^23, 60–65^.

In this study, we employed a comprehensive flow cytometric analysis of pulmonary and lymphoid cells in a mouse model of infection using a novel pandemic 2009 H1N1 (pH1N1) reporter virus in order to identify which cell types become antigen-bearing early following infection *in vivo*. We present results using the pH1N1 virus that expresses a monomeric form of Venus- a variant of the yellow fluorescent protein (YFP) that possesses several desirable features for visualization *in vivo*. Compared to parental YFP and some other green fluorescent protein (GFP) variants, the Venus coding sequence contains several amino acid substitutions (F46L, F64L, M153T, V163A, S175G, A206K) that improve its fluorescence intensity, maturation (accelerated oxidation rate of chromophore), folding kinetics and tolerance to acid and Cl^−^ ions, making it more stable in certain biological applications such as within secretory vesicles^66^. Venus was fused to the viral non-structural protein 1 (NS1), allowing for clear identification of antigen-positive cells.

These studies demonstrate that the pH1N1-Venus virus is remarkably stable for Venus expression both *in vitro* and *in vivo*, enabling sensitive detection of antigen-positive cells after infection. Flow cytometry experiments revealed that the tissue distribution and composition of antigen-bearing cells was broad and highly complex, consisting of both CD45^neg^ and CD45^pos^ bone marrow-derived cells from the lung. Multiple hematopoietic cell types, including subsets of monocytes, MΦ, DCs and neutrophils were detected and changed in representation over the course of infection. Additionally, we determined that CD103 DCs were the major CD45^pos^ cell type represented in the local dLN early during infection. Using detection of surface HA on Venus^pos^ cells as a measure of cellular infection, we found that in addition to CD45^neg^ cells, surprisingly, many different bone marrow-derived cells were infected, including DCs, aMΦ, and neutrophils. Finally, our studies revealed that many different types of influenza antigen-bearing cells express MHC class II proteins at the cell surface and differ in their capacity to re-stimulate influenza-specific CD4 T cells *ex vivo*. Taken together, these results highlight the potential of both professional (DC, MΦ) and nonprofessional (CD45^neg^, neutrophils) cells as antigen presenting cells (APC) in extra-lymphoid tissue. In summary, this work demonstrates the feasibility of using stable Venus-expressing viruses for identification of influenza-infected cells *in vivo* and has revealed the diversity of potential APC in distinct tissue microenvironments using a clinically relevant strain of significant public health importance.

## Results

### Generation of a recombinant pH1N1 virus expressing the Venus fluorescent protein

**Figure 1:**
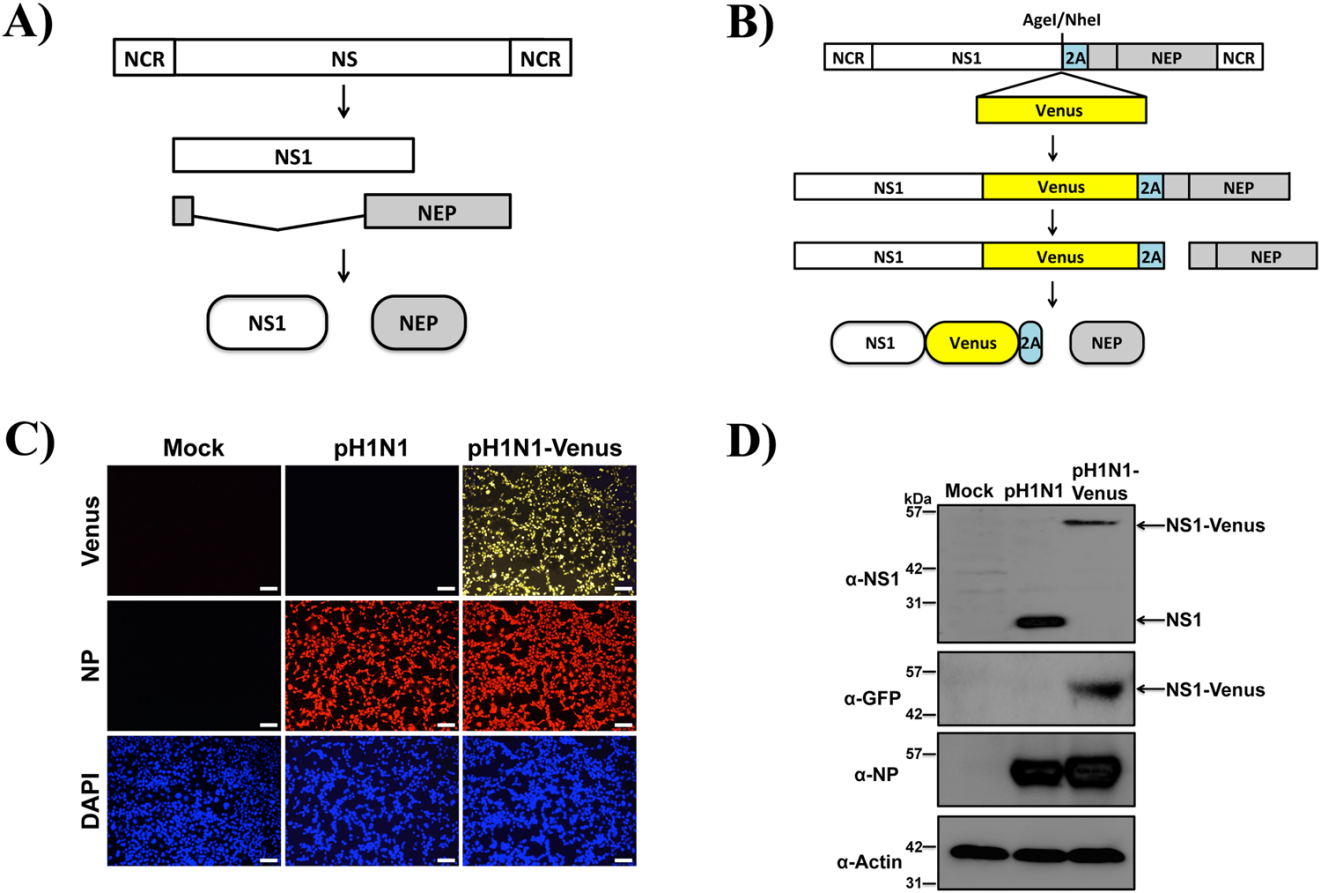
Genomic organization of NS segment and protein expression of pH1N1-Venus virus. (**A**) Schematic representation of the NS gene segment from pH1N1-WT virus, where alternative splicing of NS mRNA generates the viral NS1 and NEP proteins. (**B**) Schematic representation of the modified NS gene segment from pH1N1-Venus. The recombinant NS segment from pH1N1-Venus contains the NS1 ORF without stop codons or slice acceptor sites fused to Venus, followed by the PTV-1 2 A autoproteolytic cleavage site and the entire ORF of NEP. NCR denotes non-coding regions in the NS gene. (**C**) Immunofluoresence microscopy following infection with WT and Venus-expressing pH1N1 viruses. MDCK cells were mock infected or infected (MOI 3) with pH1N1 viruses. 18 hpi, cells were fixed and stained using an anti-NP (HB-65) antibody as well as with DAPI to visualize cell nuclei. An anti-mouse Texas-red secondary antibody was used to identify NP^pos^ cells. Representative images were captured at 10x magnification. (**D**) Western blot analysis of NS1-Venus fusion following infection. MDCK cells were mock infected or infected with WT and Venus-expressing pH1N1 viruses as described above. 24 hpi, cell lysates were analyzed by western blot as decribed in the Materials and Methods section.

To generate a replication-competent, Venus-expressing pH1N1 virus, plasmid-based reverse genetics were used^67^. It has previously been demonstrated that the NS1 protein can tolerate fusion to fluorescent proteins such as GFP and monomeric mCherry, thus making it an appropriate target for reporter gene conjugation^24, 25, 55, 56, 59^. However, because of the increased spectral, folding and acid tolerance properties, we speculated that Venus might improve sensitivity for various imaging applications including flow cytometry, confocal and multi-photon microscopy^58, 66^. Because the viral NS segment is alternatively spliced to produce NEP (Fig. 1A), two silent mutations were introduced into the splice acceptor site to prevent splicing^68, 69^. To produce NEP, the porcine teschovirus-1 (PTV-1) 2A autoproteolytic cleavage site was inserted between NS1 and NEP so that both proteins (NS1 and NEP) would be translated individually, as previously described^24, 56^. Importantly, the NS1 and NEP N-terminal overlapping ORF was duplicated downstream of the PTV-1 2A site to assure complete NEP synthesis^56^. Using two unique restriction sites (AgeI and NheI), Venus was cloned and fused to the C-terminal end of NS1 and used to generate a recombinant A/California/04/09 (H1N1) NS1-Venus virus (hereafter referred to as pH1N1-Venus) (Fig. 1B).

### Characterization of pH1N1-Venus *in vitro*

NS1-Venus protein is not packaged into virions and is only expressed following infection. Therefore, to determine Venus expression *in vitro*, MDCK cells were infected (MOI 3) with pH1N1-WT or pH1N1-Venus and imaged 18 hpi using immunofluorescence microscopy. As expected, fluorescence expression of Venus was only detectable from pH1N1-Venus infected MDCK cells, while similar amounts of nucleoprotein (NP) were observed from both infections (Fig. 1C). To demonstrate that Venus expression was due to fusion with NS1, lysates from infected MDCK cells were probed for NS1 by Western blot. Compared to the mobility pattern observed for NS1 from pH1N1-WT infection, an upward shift was observed from pH1N1-Venus infection by SDS-PAGE, indicating a larger NS1 protein complex (Fig. 1D). Furthermore, consistent with the immunofluorescence images (Fig. 1C), Venus protein expression was only detected from lysates prepared from pH1N1-Venus infected cells using a cross-reactive antibody raised against GFP (Fig. 1D). Importantly, the molecular weights were identical for species detected using the NS1 and GFP/Venus-reactive antibodies.

### Growth properties of pH1N1-Venus

**Figure 2:**
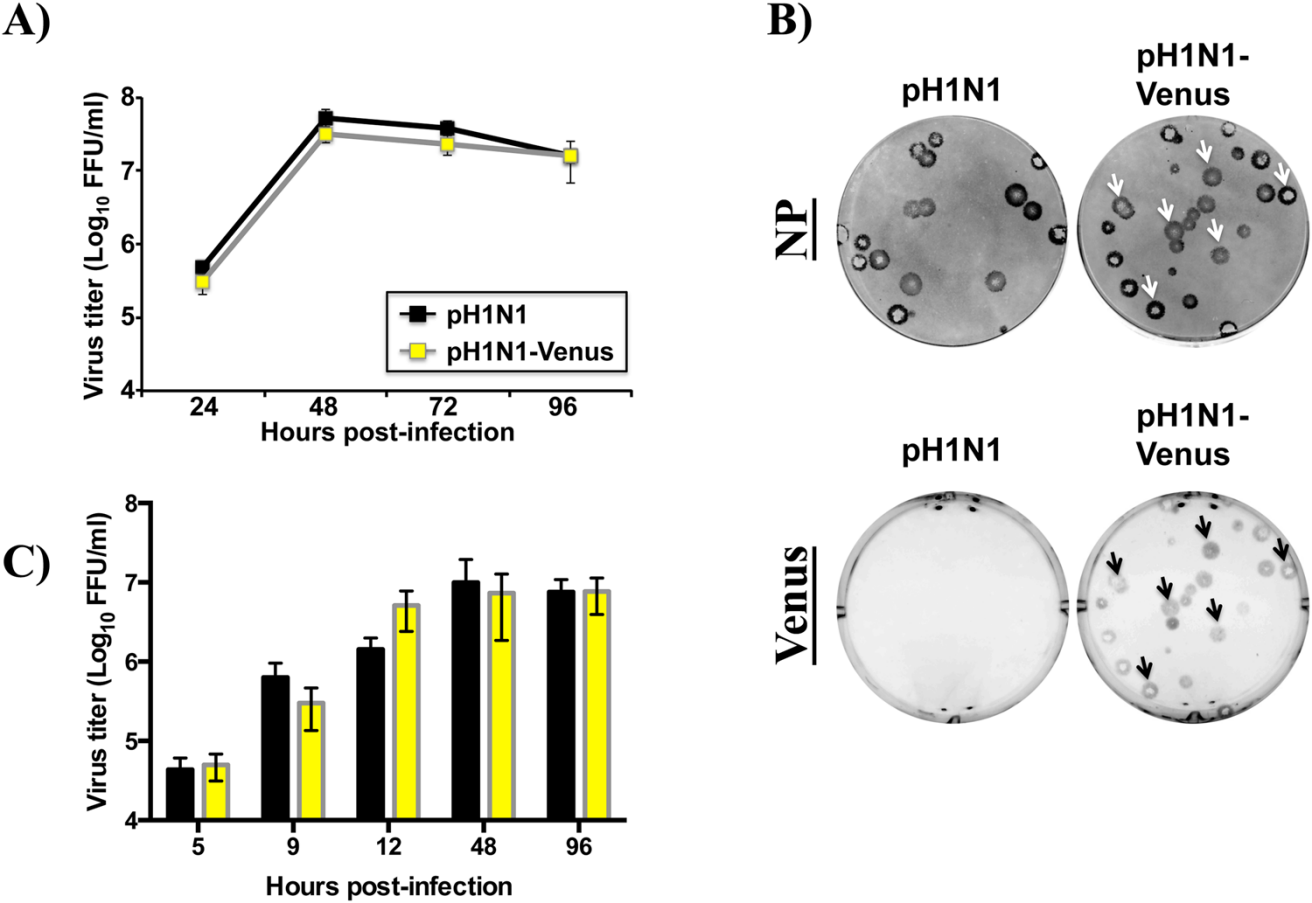
Multi-cycle growth kinetics and plaque morphology of pH1N1-Venus. (**A**) Multi-cycle growth properties of WT and Venus-expressing pH1N1 viruses. MDCK cells were infected (MOI 0.001) and tissue culture supernatants were collected at the indicated times post-infection. Viral titers in tissue culture supernatants were determined by immunofocus assay (fluorescent focus-forming units, FFU/ml) using an anti-NP (HB-65) antibody. (**B**) Plaque morphology of WT and Venus-expressing pH1N1 viruses. MDCK cells infected with pH1N1-WT and pH1N1-Venus. 3 dpi, cells were scored for NP and Venus expression as described in the Materials and Methods section. Note: arrows highlight plaques that stained positively for viral NP and NS1-Venus. (**C**) Viral replication kinetics of WT and Venus-expressing pH1N1 viruses *in vivo*. 3–4 female, C57BL/6 mice per group were infected intranasally with 10^6^ PFU virus. Lung tissue was harvested using a Dounce homogenizer at the indicated time points following infection and viral titers were determined by immunofocus assay (FFU/ml) as described in the Materials and Methods section. Error bars reflect the standard error of the mean. T-tests were performed using WT and Venus titers at each time point following infection, where p > 0.05 for all comparisons. Statistical significance was determined using the Holm-Sidak method, with alpha = 5.0%. Each timepoint was analyzed individually, without assuming a consistent standard deviation from the mean.

Virus fitness of pH1N1-Venus was next determined by evaluating the multi-cycle growth properties in cell culture using MDCK cells. Following infection, pH1N1-Venus replicated to similar titers over time compared to pH1N1-WT, suggesting that no major fitness costs were incurred in target cells of infection due to insertion of the Venus ORF (Fig. 2A). Consistent with the growth kinetics, when plaque morphology was evaluated 3 days post-infection (dpi) via immunostaining using an anti-NP antibody, plaques derived from pH1N1-WT and pH1N1-Venus infection were comparable in overall size (Fig. 2B). Importantly, when expression of Venus was evaluated, all plaques that stained positively for viral NP also were Venus^pos^, demonstrating that the pH1N1-Venus virus is genetically stable for Venus fluorescence expression during replication (Fig. 2B and Table 1). Finally, viral replication kinetics experiments *in vivo* revealed similar growth properties over time between pH1N1-WT and pH1N1-Venus (Fig. 2C), further demonstrating that the genetic modifications to the IAV genome did not result in detectable loss in virus fitness.

**Table 1:** Fluorescence stability of pH1N1-Venus *in vitro* and *in vivo*.

Virus	Titer	Fluorescence Stability^a^			
		MDCK	Lungs (C57BL/6)		
	(PFU/ml)		(3 DPI)	(4 DPI)	(5 DPI)
pH1N1-Venus	3.2 × 10^7^	147/149 (99%)	37/38 (97%)	75/75 (100%)	41/43 (95%)

^a^Fluorescence stability is reflected as a percentage, where fluorescent plaques were divided by the total number of plaques counted, and multiplied by 100.

### Tissue distribution of Venus^pos^ cells following infection *in vivo*

**Figure 3:**
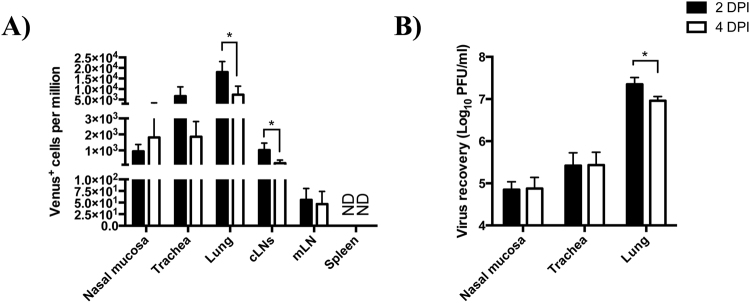
Tissue distribution and pH1N1-Venus virus recovery following infection *in vivo*. (**A**) Female, C57BL/6 (I-A^b^) mice were infected with 10^6^ PFU pH1N1-Venus. Respiratory (nasal mucosa, trachea and lung) and lymphoid (cLNs: cervical lymph nodes, mLN: mediastinal lymph node and spleen) tissues were enzymatically and mechanically dissociated to prepare single cell suspensions and live, single cells were sampled for Venus expression via flow cytometry 2 and 4 dpi. ND stands for Not Detectable (**B**) Virus recovery from infected respiratory tissues following pH1N1-Venus infection. Supernatants from pelleted cells were reserved from infected tissues and used as the source of virus for quantification via plaque assay at the indicated time points. *denotes statistical significance as determined by students t test, where p < 0.05. Responses from 4 individual animals per group were used for statistical analysis.

Influenza virus infections are initiated and propagated in the respiratory tract. During the course of infection, pulmonary cells have the potential to access viral antigens by different mechanisms. Cells can either become infected or take up viral antigen(s) exogenously, typically from dying cells or after release of viral proteins. While some antigen-positive cells are migratory, many others remain resident to the site of viral replication and serve to eliminate virus either directly or indirectly through recruitment of additional immune cells. While some resident cells such as MΦ and neutrophils die in response to viral infection or after performing their effector function, other cells may persist as sources of late antigen reservoirs and play important roles in regulating adaptive immunity^70, 71^. We therefore sought to characterize the tissue and cellular distribution of antigen-bearing cells early following infection *in vivo* using pH1N1-Venus. To this end, C57BL/6 (I-A^b^) mice were infected intranasally with 10^6^ PFU pH1N1-Venus virus and respiratory and lymphoid tissues were harvested 2 and 4 days later. Single cell suspensions were generated from enzymatic and mechanical tissue disruption and single, live cells were evaluated for Venus expression by flow cytometry. These experiments revealed that Venus^pos^ cells were widely distributed throughout the mouse respiratory tract 2–4 dpi, where lung (≈12,000 cells per million) > trachea (≈4,000 cells per million) > nasal mucosa ≈1,300 cells per million) (note: cell frequencies reflect the average taken from 2 and 4 dpi) (Fig. 3A). Plaquing of virus and visualizing Venus expression from virus recovered from cell supernatants derived from the respiratory tract (Fig. 3B) indicated that the pH1N1 reporter virus was remarkably stable for Venus expression (Table 1), and that the initial source of viral antigen was derived from viral infection. While Venus^pos^ cells could not be detected in the spleen, they were found in other secondary lymphoid tissues draining the upper (cervical lymph nodes, cLN) and lower (mediastinal lymph nodes, mLN) airways, albeit in very low quantities (Fig. 3A).

### Immunophenotype and cell distribution of Venus^pos^ cells in the infected lung

**Figure 4:**
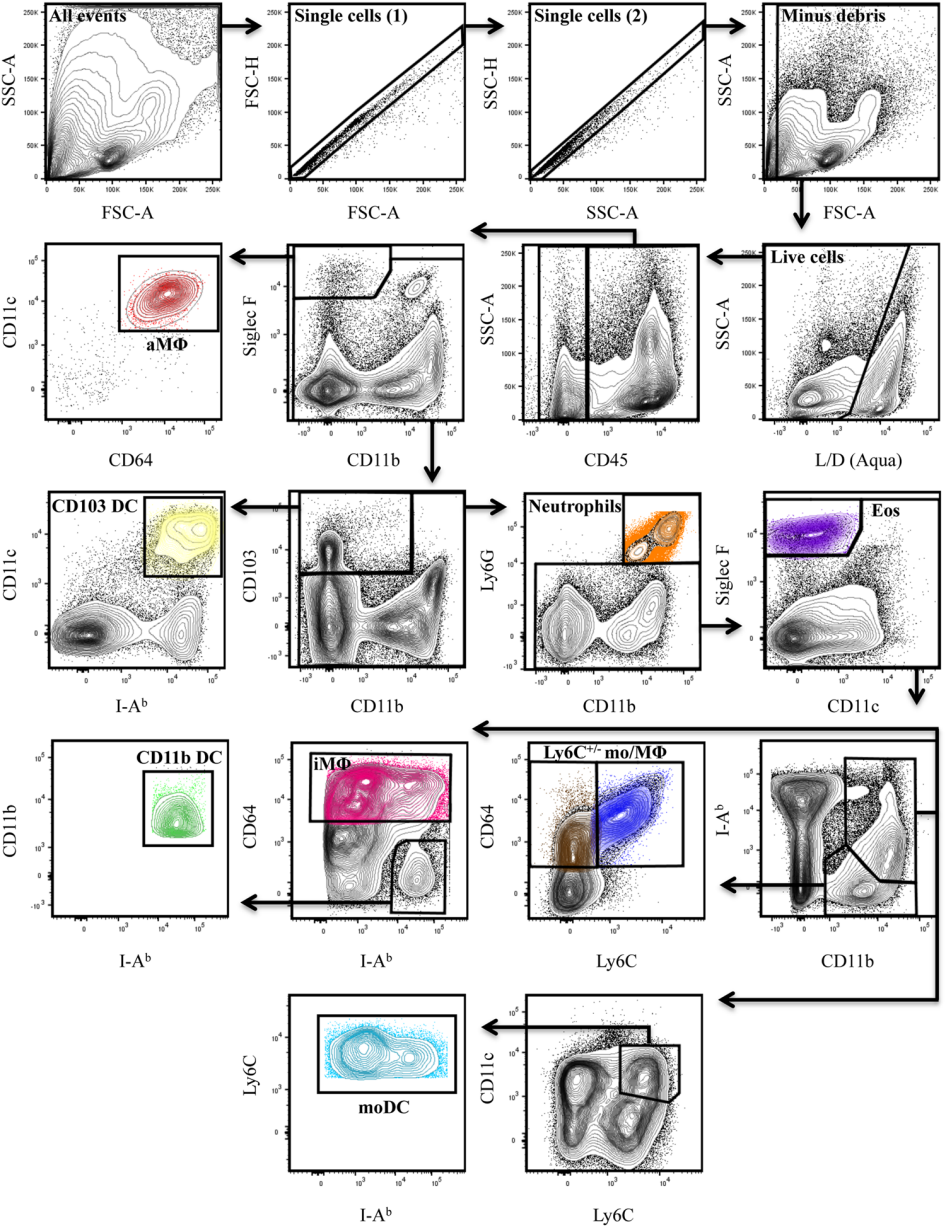
Flow cytometry gating strategy used to identify cell subsets following pH1N1-Venus infection. After infection of mice with pH1N1-Venus, cells were enzymatically and mechanically dissociated to produce single cell suspensions. Using a sequential gating strategy, bone marrow- derived cells were distinguished on the basis of CD45 expression and antibodies to specific markers (as outlined in Table 2) were used to identify alveolar macrophages (aMΦ) (Siglec F^+^CD11b^−/lo^CD64^+^CD11c^+^), CD103 DC (CD103^+^CD11c^+^I-A^b+^), eosinophils (Siglec F^+^CD11b^+^CD11c^−^) and neutrophils (Ly6G^+^CD11b^+^). Cells with overlapping expressions were identified as follows: interstitial macrophages (iMΦ) (CD11b^+^I-A^b+^CD64^int/hi^), CD11b DC (CD11b^+^I-A^b+^CD64^−^), monocyte-derived DC (moDC) (CD11b^+^I-A^b+^Ly6C^+^) and monocytes/undifferentiated macrophages (mo/MΦ) (CD11b^+^I-A^b^ ^-/lo^CD64^+^Ly6C^+/−^). Note: representative panels derived from an infected lung sample (2 dpi).

**Figure 5:**
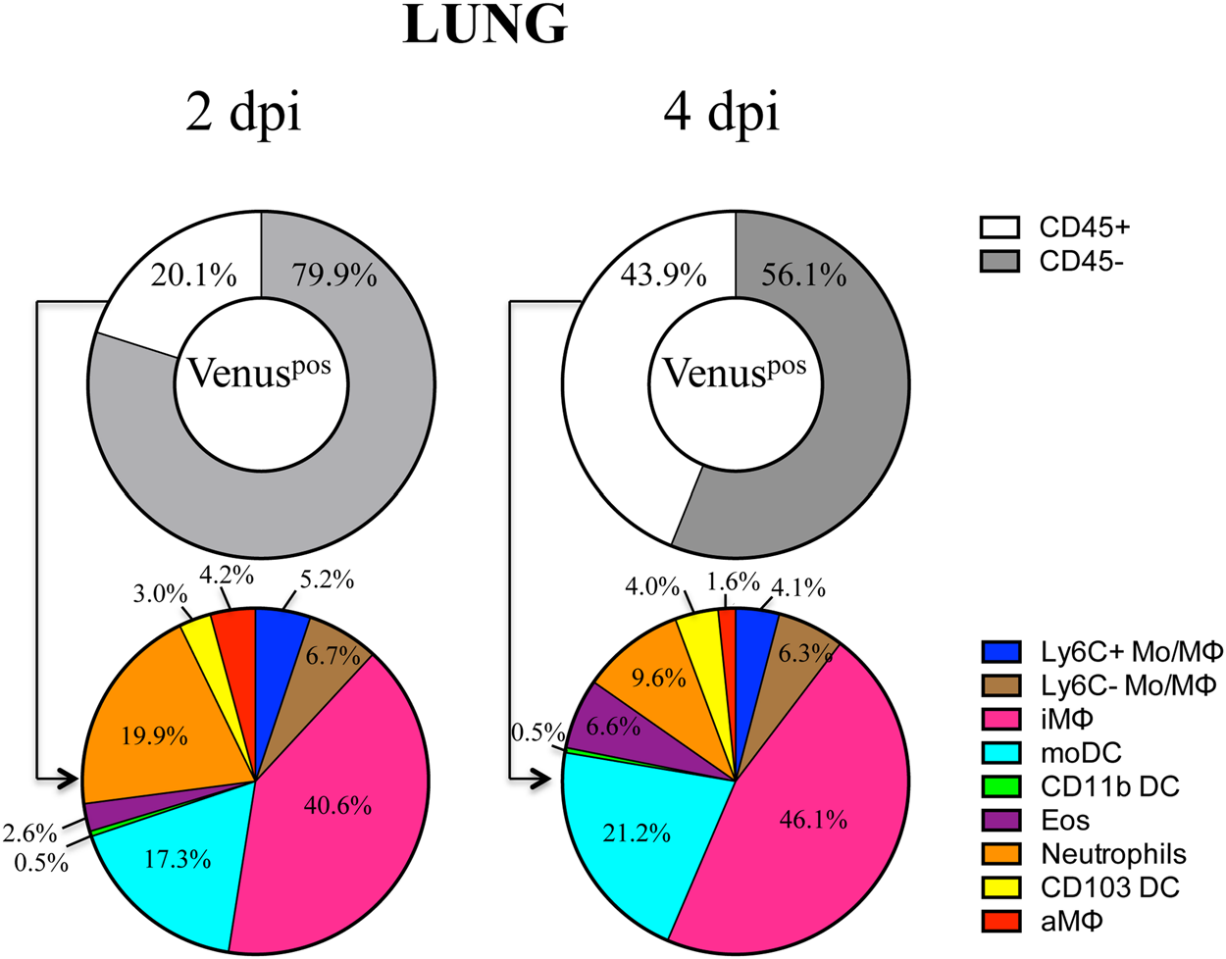
Distribution of Venus-bearing cells in the lung following infection. Composition of Venus^pos^ cells 2 and 4 dpi with 10^6^ PFU pH1N1-Venus virus per mouse. Pie charts represent a composite from two independent experiments, with responses pooled from 5 mice per group.

The lung and local dLN are highly heterogeneous in cell composition, consisting of hematopoietic (CD45^pos^, bone marrow-derived) and non-hematopoietic (CD45^neg^) cells with the ability to access and handle influenza antigens following viral infection. To identify as many distinct cell types as possible with the potential to become antigen-bearing following infection, we took advantage of recent progress in multi-parameter flow cytometry analyses of lung tissue under inflamed conditions^33, 34, 72^. Mice were infected with pH1N1-Venus and a combination of previously described antibodies (Table 2) and gating schemes (Fig. 4) for the identification of a variety of myeloid cells was used to characterize the phenotype of NS1-Venus^pos^ cells early post-infection (2 and 4 dpi) in the lung (Fig. 5)^33, 73^.

**Table 2:** Surface marker expression by cell type.

	Marker	CD103 DC	CD11b DC	moDC	aMΦ	iMΦ	Ly6C^+^ Mo/MΦ	Ly6C^−^ Mo/MΦ	Eosinophils	Neutrophils
1	CD45	+	+	+	+	+	+	+	+	+
2	CD11b	−	+	+	−	+	+	+	+	+
3	Siglec F	−	−	−	+	−	−	−	+	−
4	CD11c	+	+	+/−	+	+	−	+/−	−	−
5	CD64	−	−	+	+	+	+/−	+/−	−	−
6	I-Ab	+	+	+/−	+	+	−	−	+/−	+
7	CD103	+	−	−	−	−	−	−	−	−
8	Ly6-G	−	−	−	−	−	−	−	−	+
9	Ly6-C	−	+/−	+	−	−	+	−	−	+/−

+/− denotes low/intermediate or inducible expression.

Figure 4 illustrates the gating scheme and Table 2 summarizes the biomarker expression profiles used to resolve Venus-positive cell populations. Multi-parameter flow cytometry has proven to be essential for the resolution of different cell types in the lung after infection. Overlapping expression profiles between cell types and some alterations in marker expression within individual populations following infection has complicated efforts to identify distinct cell types and their origins. As a result, some differences regarding the selection of cell surface markers and their antigen density has been reported. Therefore, the cell types described below were operationally identified using information gathered from recent studies^10, 11, 33, 34^. To simplify nomenclature, cell types will hereafter be referred to by their population name and not their marker expression profile. First, single, live cells were selected based on their ability to exclude staining of a viability dye, in combination with forward and side scatter features. Non-hematopoietic cells (epithelial, stromal and endothelial cells) were distinguished from hematopoietic cells based on CD45 expression. Cell types that express highly specific markers such as aMΦ (Siglec F^+^CD11b^−/lo^CD64^+^CD11c^+^), CD103 DC (CD103^+^CD11c^+^I-A^b+^), eosinophils (Siglec F^+^CD11c^−^CD11b^+^) and neutrophils (Ly6G^+^CD11b^+^) were then identified. Cells with a higher degree of marker overlap were identified as follows: iMΦ (CD11b^+^I-A^b+^CD64^int/hi^), CD11b DC (CD11b^+^I-A^b+^CD64^−^), moDC (CD11b^+^I-A^b+^Ly6C^+^) and mo/MΦ (CD11b^+^I-Ab^−/lo^CD64^+^Ly6C^+/−^). Venus^pos^ cells were identified for each cell type based on gates that were drawn from cells obtained by non-Venus WT infected mice (Supplemental Fig. 1), which were equivalent in cell composition (Supplemental Fig. 2) and populations identified using the same set of antibodies specific to the markers illustrated in Fig. 4 and summarized in Table 2.

These analyses revealed that the majority of Venus^pos^ cells in the lung were CD45^neg^ (68 ± 12%). However, there was a significant gain in the frequency of CD45^pos^ cells over time (20.1→43.9%), consisting of many different cell types, including monocytes, macrophages (aMΦ, iMΦ), DCs (CD103^+^, CD11b^+^, Ly6C^+^ moDC), neutrophils and eosinophils (Fig. 5). iMΦ and blood-derived moDC accounted for the majority of Venus^pos^ cells, which increased slightly in representation over time (57.9→67.3%). During the course of infection, the distribution of some cell types was altered. For example, decreases in the percentages of neutrophils (19.9→9.6%) and aMΦ (4.2→1.6%) were observed between 2–4 dpi, with a concomitant increase in eosinophils (2.6→6.6%). Moreover, other cell types, including conventional DCs (cDCs) (i.e. CD103^+^ and CD11b^+^ subsets) and Ly6C^+/−^Mo/MΦ remained relatively unchanged in terms of their fractional response. Reproducible quantification of Venus^pos^ cells was also possible using a 10-fold lower dose (10^5^ PFU/mouse), which importantly, did not alter the diverse cellular composition of antigen-bearing cells in the lung (Supplemental Fig. 3). Collectively, our results revealed that many distinct cell types gain access to NS1-Venus antigen. The differences in cell distribution over time may reflect cell turnover, influx and/or egress of cells from the tissues.

**Figure 6:**
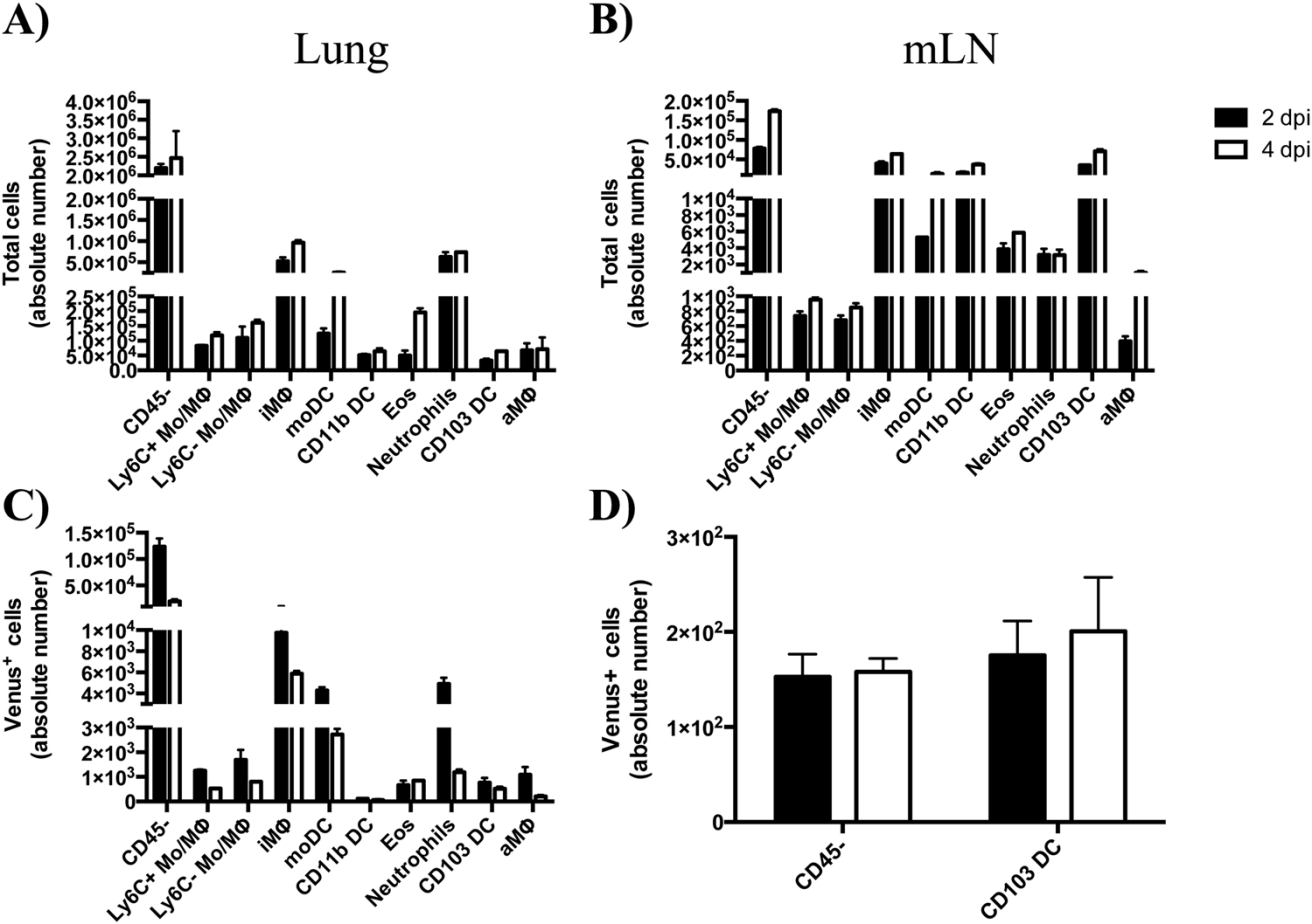
Cell dynamics in lung and LN following infection. (**A**,**B**) Comparison of absolute cell number by tissue, time and cell type following infection. (**C**,**D**) Comparison of Venus^pos^ cell number by tissue, time and cell type following infection. Bar graphs represent the average from two independent experiments (5 mice per group) and reflect the average number of cells per animal. Error bars denote range. Mice were infected with 10^6^ PFU pH1N1-Venus.

**Figure 7:**
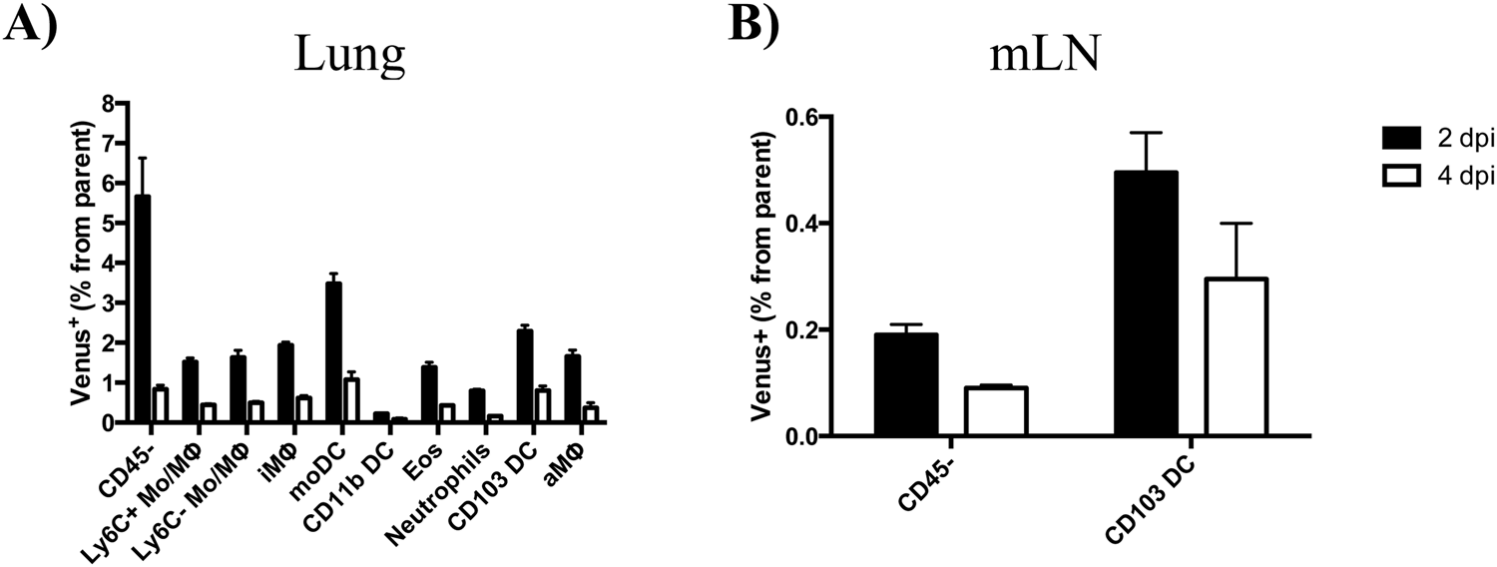
Percentage of antigen-bearing cells by cell type, tissue and time following infection. Bar graphs represent the average from two independent experiments, with responses pooled from 5 mice per group. Error bars denote range. Mice were infected with 10^6^ PFU pH1N1-Venus.

To further study the multiple cell populations, we analyzed the total abundance of each cell type (Fig. 6A,B) as well as those that were Venus^pos^ (Fig. 6C,D) to determine if any cell types were preferentially enriched in antigen-bearing cells. Venus^pos^ cell populations were most abundant 2 dpi in the lung (Fig. 6C) and showed very good agreement with the frequency of each cell type (Fig. 7). While we were able to identify cells in the draining lymph node with similar surface marker expression profiles to those in the lung (Fig. 6B), only a very low frequency of Venus^pos^ cells were identifiable in this tissue. As shown in Fig. 6D, fewer than 1,000 Venus^pos^ cells were quantified per mouse. However, we were able to distinguish CD45-negative (35–38%) and positive (62–65%) cells and found that the majority from the CD45^pos^ lineage (>90%) had markers associated with CD103 DC 2–4 dpi (Supplemental Fig. 1B).

### Venus and cell-surface HA expression identifies infection status of cells following infection

**Figure 8:**
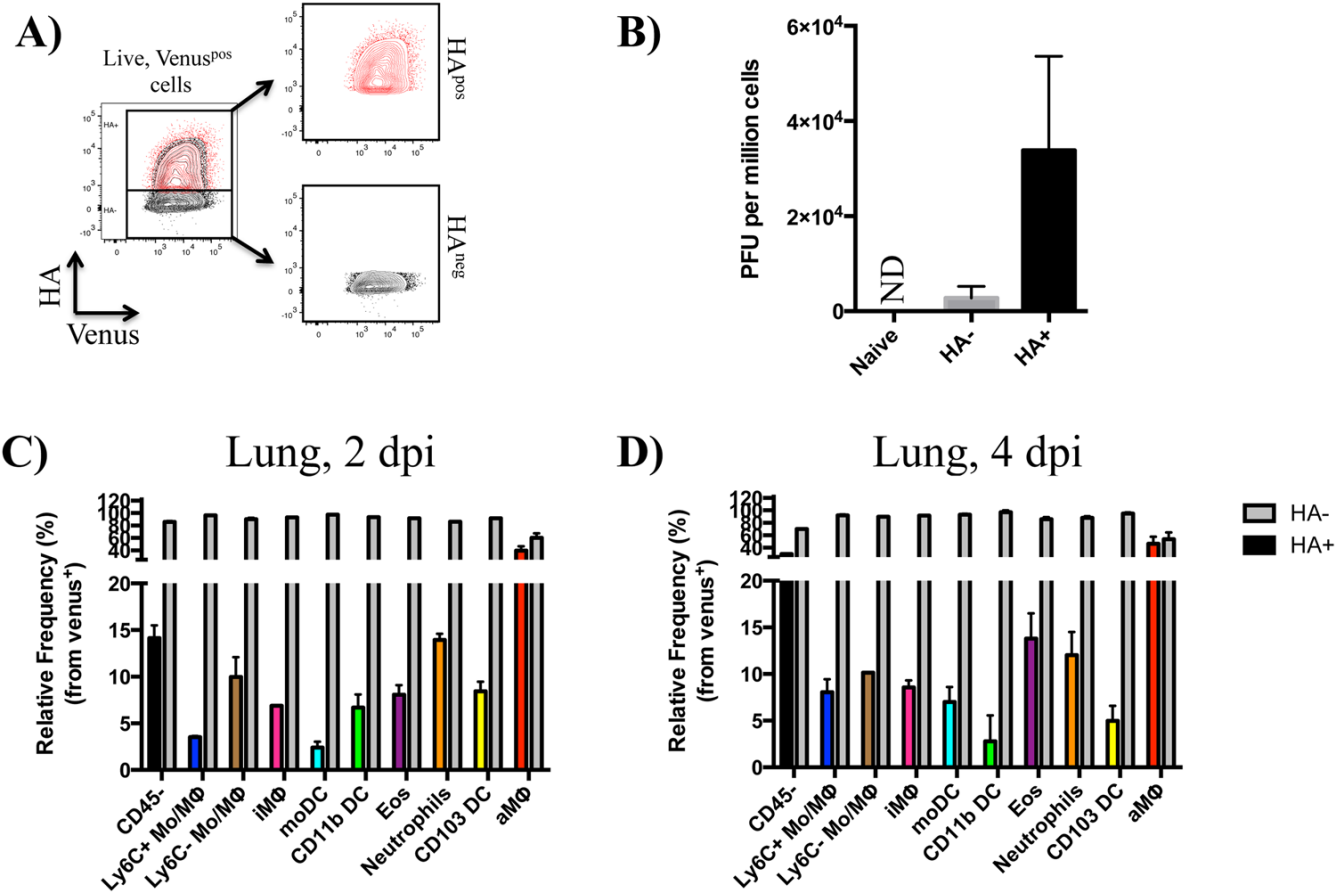
Venus and HA expression enrich for virus-producing cells and varies by cell type in the infected lung. (**A**) FACS gating scheme for purification of Venus^pos^/HA^neg^ and Venus^pos^/HA^pos^ cells 2–3 dpi from the lungs of mice infected with 10^6^ PFU pH1N1-Venus virus. (**B**) Virus recovery from Venus^pos^/HA^neg^ and Venus^pos^/HA^pos^ sorted cells. MDCK cells were infected with the sorted cell populations in dilution series for 3 hours at 33 °C. Cells were then washed and virus yield was determined by plaque assay. Bar graphs represent the average responses from two independent experiments using 3–4 mice per group, 2–3 dpi. Error bars denote the range. ND stands for Not Detectable. (**C**,**D**) Surface HA expression by cell type, 2 and 4 dpi in the lung. Grey bars represent the frequency of HA^neg^ cells and colored bars represent the frequency of HA^pos^ cells, pregated on Venus^pos^ cells. Data for each cell type reflects the fractional response, where the sum of HA^neg^ and HA^pos^ equals 100 percent. Bar graphs represent the average from two independent experiments, with responses pooled from 5 mice per group. Error bars denote range.

The preceding analyses revealed that many different cell types appear to access influenza antigen. However, the route by which this occurred in different subpopulations was not evaluated in the previous experiments. This is an important area to resolve because the modes of antigen acquisition (infection versus protein uptake) will have different consequences in initiating the adaptive response and the display of peptide:MHC complexes^38^. To distinguish infection from exogenous protein uptake on antigen-bearing cells, we evaluated surface expression of the viral HA protein^30^ on cells that were Venus^pos^ as a measure of virus infection (Fig. 8 and Supplemental Fig. 4)^74^. Therefore, single, live Venus^pos^ cells from the infected lung (2–3 dpi) were sorted on the basis of surface HA expression, detected with a MAb to pHA^75^ using FACS as depicted in Fig. 8A. Approximately 1–3% of all live cells were Venus^pos^ in the lung 2–3 dpi. Two discrete populations were resolved on the basis of surface HA expression, where approximately 15% were HA^pos^ and 85% were HA^neg^. Compared to the HA^neg^ population, we found that the HA^pos^ population was enriched in productively infected cells, as measured by their ability to infect MDCK cells in a co-culture *ex vivo* and quantified by plaque assay (Fig. 8B). We considered the possibility that a subset of the HA^neg^ cells had actually undergone infection but were kinetically delayed in cell surface accumulation of HA. Therefore, Venus^pos^/HA^neg^ cells were cultured for 16 hours and re-stained with anti-HA antibody to test whether some cells gained membrane-associated HA (Supplemental Fig. 5). Because there was not a detectable gain in surface HA over this extended time frame, we conclude that the HA^neg^ cells gain access to NS1-Venus by uptake of exogenous viral antigen.

To better define the populations identified earlier based on HA expression, the frequency of HA^pos^ cells was quantified for each Venus^pos^ cell type in the lung 2 and 4 dpi. Although the majority of Venus^pos^ cells were HA^neg^, all of the cell types identified contained a fraction of cells that were HA^pos^, ranging from approximately 5–60%, depending on the population early following infection (Fig. 8C,D). Ly6C^+/−^ monocytes/undifferentiated MΦ (7.9%), iMΦ (7.7%) and DCs (3.6%) contained the lowest frequencies of HA expression, while CD45^neg^ (22%), neutrophils (13%), eosinophils (11%) and aMΦ (43%) contained the highest (note: cell frequencies reflect the average from 2 and 4 dpi). Interestingly, while some cell types (aMΦ and neutrophils) expressed relatively stable frequencies of HA^pos^ cells between 2–4 dpi, other populations decreased (migratory cDCs) and others gained (eosinophils, moDC as well as CD45^neg^ cells) HA^pos^ cells over time. Due to the low frequency and number of Venus^pos^ cells in the mLN, we were unable to clearly discern if any cells were HA^pos^.

### Venus^pos^ cells vary in MHC class II expression and can stimulate influenza-specific CD4 T cells

Professional APCs such as DC and MΦ constitutively express MHC class II. Other cells, such as epithelial cells and monocytes can induce or upregulate class II molecules in response to Toll-like Receptor (TLR) engagement and cytokines such as IFN-γ. Any of the cells that are antigen-bearing and express class II molecules are potential targets of CD4 T cell recognition. Within the lung, MHC class II-positive cells that are antigen-bearing may serve as targets of CD4 tissue-resident memory cells established by previous infections, where CD4 T cells might cause cytolysis of infected cells^76–79^ or direct cytokine mediated events important for recruitment of other cells into the lung^15, 16^. Some class II-positive cells, such as DCs, exit the lung and migrate to the dLN in a CCR7-dependent manner, where antigen is presented to T and B lymphocytes^80^. In the dLN, antigen can be presented either directly or is transferred to resident cells (e.g. CD8αα DC) for additional handling, ultimately leading to lymphocyte activation and expansion (reviewed in ref. 22). Additionally, later in the response, other possible APC in the tissue (e.gs., endothelial cells, epithelial cells, eosinophils and neutrophils) might play a role in providing instructive cues for further differentiation, retention or maintenance of memory CD4 T cells^81, 82^. Because class II is much more restricted than MHC class I molecules (expressed by all nucleated cells), it was important to evaluate the class II expression profiles of Venus^pos^ cells in the lung.

**Figure 9:**
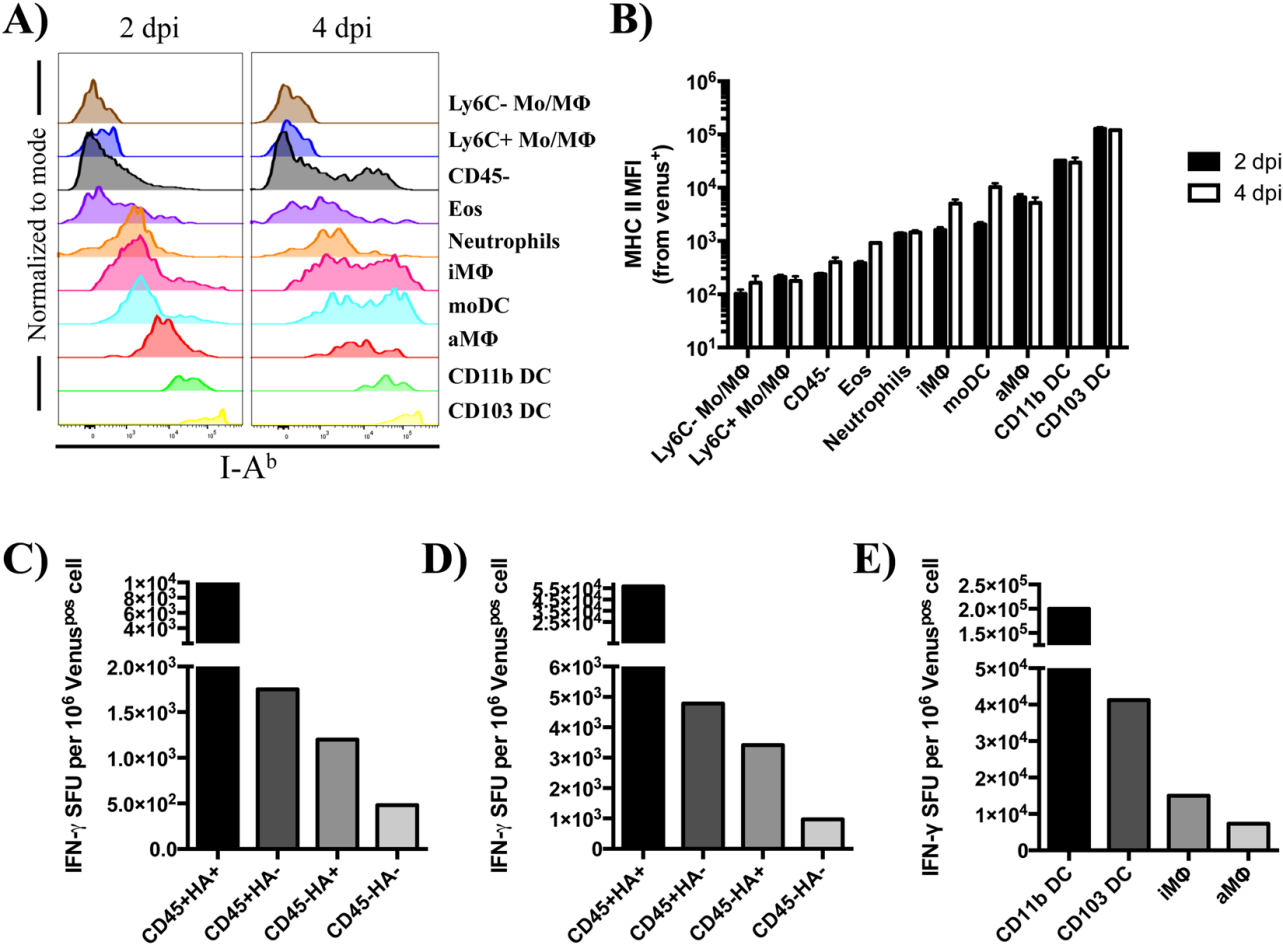
Venus^pos^ cells vary in MHC class II expression and can stimulate influenza-specific CD4 T cells. (**A**) Histograms representing class II (I-A^b^) expression profiles on Venus^pos^ cells, 2 and 4 dpi in the lung. (**B**) Median fluorescence intensity (MFI) of class II by cell type. Bar graphs represent the average from two independent experiments using 5 mice per group and error bars denote range. (**C**–**E**) Frequency of IFN-γ-producing, influenza-specific CD4 T cells that were re-stimulated following co-culture with Venus^pos^ cells derived from infected lung tissue (2 dpi). Note: Data presented in panels (C,E) reflect the number of IFN-γ spot forming units (SFU) from 55,000 and 75,000 antigen-experienced CD4 T cells, respectively. Data presented in panel (D) utilized 75,000 CD4 T cells that were expanded for 12 days in culture using a combination of peptides and recombinant IL-2 protein (*see Materials and Methods*). OVA-specific CD4 T cells were elicited and tested in parallel as a specificity control, which generated no IFN-γ SFU when co-cultured with Venus^pos^ cells isolated from influenza-infected animals. Bar graphs represent the mean from two technical replicates in the assay. For cell sorting, the following CD11c + subpopulations were identified as follows: CD64^+^CD24^−^ macrophages, aMΦ (Siglec F^+^CD11b^−^), iMΦ (Siglec F^−^CD11b^+^) and CD64^−^CD24^+^ DCs, CD103 DC (CD103^+^CD11b^−^), CD11b DC (CD103^−^CD11b^+^).

MHC class II expression profiles of Venus^pos^ cells are shown in Fig. 9A. Compared to Ly6C^+/−^ Mo/MΦ, which express little or no MHC class II molecules, in agreement with earlier studies^33^, a wide range of expression for other cell types was found, with unimodal and bimodal distributions that varied sharply in fluorescence intensity. For instance, cDCs were uniform in distribution and displayed the highest MFI (≈78,000) (Fig. 9B). Somewhat unexpectedly, we found that neutrophils and lung resident macrophages (aMΦ and iMΦ) uniformly expressed intermediate levels of class II molecules (≈6,000 and 3,500 MFI, respectively). Furthermore, some cell types such as iMΦ, moDC and CD45^neg^ cells displayed a bimodal distribution, with an increased frequency of cells expressing higher median fluorescence intensity over time.

To test whether Venus^pos^, antigen-bearing cells had the capacity to stimulate CD4 T cells, we isolated them based on differential expression of CD45 and HA using FACS, followed by co-culture with influenza-specific CD4 T cells, generated by *in vivo* peptide priming. We hypothesized that if Venus^pos^ cells displayed viral peptides bound to MHC class II molecules, they could have the ability to re-stimulate the *in vivo*-primed, influenza-specific CD4 T cells to secrete IFN-γ, which can be captured and measured in an ELISpot assay, optimized to quantify antigen-bearing cells. Here, a lawn of antigen-specific CD4 T cells are plated in excess, with limiting numbers of the different populations of candidate APC that may display viral peptide:MHC class II complexes at the cell surface. IFN-γ is then captured and quantified as previously described^83–85^. In two independent experiments (Fig. 9C,D), we found that all four subpopulations of Venus^pos^ cells could re-stimulate the CD4 T cells to produce antiviral cytokine, and thus display viral peptide(s) presented by MHC class II molecules. Interestingly, the percentages of each Venus^pos^ cell type capable of eliciting cytokine differed considerably, where CD45^+^HA^+^ > CD45^+^HA^−^ = CD45^−^HA^+^ > CD45^−^HA^−^, corresponding to 4, 0.4 0.5, and 0.01%, respectively. Importantly, although CD45^+^HA^+^ represented only 2% of all Venus^pos^ cells (10% of all bone marrow-derived cells), they were the most potent among the four cell types and were approximately 10, 8 and 400-fold more potent on a per cell basis than CD45^+^HA^−^, CD45^−^HA^+^, CD45^−^HA^−^ cells, respectively.

Because the antigen-bearing CD45^+^HA^+/−^ populations are comprised of CD11c^+^ cells from dendritic cell and macrophage lineages, we next determined which major cell type(s) were responsible for epitope display. These studies revealed that again, each CD11c^+^ cell type studied (cDCs, aMΦ and iMΦ) was able to elicit CD4 T cell-dependent cytokine, where CD11b DC (~20%) > CD103 DC (~4%) > iMΦ (~2%) = aMΦ (~1%), with the number in parenthesis indicating the percentage of cells capable of stimulating the *in vivo* primed, polyclonal CD4 T cells (Fig. 9E). Based on these experiments, we estimate that cDCs, representing between 0.5–5% antigen-bearing CD45^+^ cells in the lung 2 dpi were up to ~20 times better APCs in culture compared to the more highly abundant macrophages (4–40% of the CD45^+^ cells) on a per cell basis, with possible differences between DC subsets. As particular subsets of respiratory dendritic cells (e.g. cDC) are mobilized within the lung and undergo accelerated movement to the draining mLN soon after infection where they are thought to prime CD4 and CD8 T cells^9, 30, 86, 87^, the total frequency of DCs detected in the lung is likely an underestimate. Collectively, this data highlights the potential of a wide range of cell types that have the potential to form contacts with CD4 T cells following infection

## Discussion

In this study, we sought to identify the key cell types that become antigen-bearing early following influenza virus infection *in vivo*. To this end, we used a fluorescent reporter virus (Fig. 1) that enabled detection of NS1-Venus^pos^ cells in a mouse model of infection in conjunction with multi-parameter flow cytometry (Fig. 4). These studies revealed that the cell composition of antigen-positive cells was highly diverse in the lung, including CD45^neg^ and CD45^pos^ cells and changed over time (Fig. 5). We suspect that the majority of Venus^pos^ CD45^neg^ cells were airway epithelial cells, which are known to be the major target cells of influenza virus infection. The Venus^pos^ CD45^pos^ bone marrow-derived cells from the infected lung consisted of many cell types, including surface marker expression profiles consistent with monocytes, MΦs, cDCs, neutrophils and eosinophils Throughout the course of infection (2–4 dpi), iMΦ, moDC and neutrophils accounted for approximately 80% of CD45^pos^ Venus^pos^ cells in the lung, while CD103 DC were the only bone marrow-derived cell type in the dLN that was readily detectable under the experimental conditions described in this report. We also observed dynamic changes in representation for other antigen-bearing cells in the lung over time that likely reflects a combination of events, including influx or egress, cell death or cell proliferation.

Previous studies that provided the first glimpse of influenza infection, while exciting, were limited in several respects. Earlier studies characterizing the PR8 (Mt. Sinai) NS1-GFP system revealed that the reporter virus was less stable for fluorescence expression over time *in vitro* and *in vivo* ^24^, indicating that after replication, infection and antigen handling were likely to be less detectable due to the loss of GFP expression. Together, the success reported here in identification of multiple antigen-bearing cell types (especially MΦ and DCs) was possibly enhanced by a combination of improved fluorescence stability and the strain (pH1N1) of virus used. Additionally, recent technical advances in defining reliable biomarkers allowed for better discrimination between MΦ and DC subsets (e.g., CD11b DC from iMΦ), revealing that iMΦ account for a significant portion of Venus^pos^ cells in lung tissue.

Using a monoclonal antibody to the viral HA, we found that there were two readily detectable Venus^pos^ cell populations that differed qualitatively in expression of HA (Fig. 8). Of the total Venus^pos^ cells, approximately 15% were also HA^pos^. Venus^pos^/HA^pos^ cells were highly enriched for virus-producing cells, relative to the HA^neg^ population, likely reflecting that only this population contains productively infected cells. Interestingly, all cell types measured in this study contained some HA^pos^ cells, indicating that all were susceptible to infection by this H1N1 virus. Within the Venus^pos^/HA^pos^ population, we found that although CD45^neg^ non-bone marrow-derived cells were the most abundant, CD45^pos^ aMΦ, and neutrophils contained equivalent or higher frequencies of Venus^pos^/HA^pos^ cells. This result suggests that aMΦ and neutrophils may either be more susceptible to viral infection and/or be positioned in locations within the lung microenvironment that enable them better access to infectious virions.

Our observation that Venus^pos^/HA^pos^ cells are derived from many different cell types, particularly those derived from hematopoiesis (CD45^pos^) is important because of the possible consequences on the outcome of infection. Evidence suggests that bone marrow-derived cells can respond differently to viral antigens, in part based on the context of antigen (virus versus antigenic fragments) and recognition of pathogen-associated molecular patterns (PAMPS) and damage-associated molecular patterns (DAMPS) by cytosolic and/or endosomal innate sensors (reviewed in refs 8, 17 and 88). Integration of these multiple signaling events culminate in the production and release of type I and III IFNs, proinflammatory cytokines and chemokines that together coordinate potent antiviral immune responses (reviewed in refs 9, 14 and 89).

For example, CD11b DCs are heterogeneous and differentially express pathogen recognition receptors (PRRs) such as TLR7, the receptor for single-stranded RNA^9^. Following ligation through PRRs, a variety of proinflammatory chemokines such as MCP-1, MIP-1α and RANTES and other mediators (e.gs., TNF and iNOS) are produced that have been shown to contribute to T cell activity as well as influenza pathogenesis and disease^90, 91^. In contrast, other cells such as nonconventional DCs (e.g., plasmacytoid DC) produce different mediators in response to TLR 7 signaling, such as type I IFN, and have been documented to play different roles in immunity. These include enhancing the influenza specific antibody response through provision of type I IFN and IL-6, altering the balance between Th1 and Th2 cells through polarization of Th1 via type I IFN and IL-12 and modulating cDC maturation^9^.

These studies also revealed that many of the Venus^pos^ cells also expressed MHC class II molecules (Fig. 9). Thus, these cells have the potential to interact with influenza virus-specific CD4^+^ cells. Here, the different routes of antigen acquisition may bias both the quality and magnitude of the adaptive response. For instance, infection may result in higher protein content due to active viral protein synthesis and influence peptide:MHC cell surface densities, potentially modulating signaling threshold requirements and effector programming. Furthermore, infection compared to exogenous protein uptake may skew the viral protein composition within endocytic compartments. Uptake of dead cells or shed viral antigen would be expected to localize all of the internalized antigen within the endosomal/lysosomal class II processing compartments. Our studies revealed that after infection, both CD45-negative and positive cells from the lung display sufficient peptide:class II complexes to be recognized and elicit cytokine from a polyclonal population of influenza-specific CD4 T cells. This suggests that different subpopulations of antigen-presenting cells in the lung may be available to encounter tissue resident CD4 T cells established from previous infections or facilitate the recruitment of additional virus-specific CD4 T cells from circulation.

Importantly, we have identified and confirmed that multiple Venus^pos^ cell subsets in the lung can stimulate influenza-reactive CD4 T cells (Fig. 9C,E), which appeared to differ based on infection status, lineage and MHC class II molecule expression. For example, we found that independently of CD45 expression, infected cells were more potent APCs compared to uninfected cells. However, of the CD45^+^ cells, cDCs were better APCs compared to macrophages and this result positively correlated with the degree of MHC class II expression, which has been shown to play an important role in facilitating T cell effector responses^92^. We speculate that the lower potency of CD45^−^ cells and CD45^+^ macrophages may have been due to one or more of the following factors. First, it is possible that different cell types access different antigenic mixtures and may not present antigenic peptides equally. Second, different cell types may possess different intracellular antigen processing machinery as well as key regulators of peptide:MHC class II presentation such as invariant chain, HLA-DM and HLA-DO. It is conceivable that such differences could ultimately result in lower densities of peptide:class II complexes necessary for CD4 T cell recognition for distinct cell types^93, 94^. Alternatively (or additionally), different cell types that undergo maturation as a result of antigen encounter in the lung may upregulate and/or downregulate different co-stimulatory and regulatory markers^95–98^. It is possible that an imbalance of such signals at the cell surface may diminish the potency of cytokine secretion by stimulated CD4 T cells *ex vivo*. While we find it intriguing that a subset of CD45^neg^ in the lung-draining lymph node were Venus^pos^ (Figs 6D and 7), very low numbers precluded our ability to assay for antigen presentation in this tissue in parallel. Therefore, at this time, we can only speculate that the CD45^neg^ cells may include follicular dendritic cells, fibroblastic reticular cells, marginal reticular cells and/or migrant epithelial cells from the lung^25, 99, 100^. Furthermore, it is possible that some Venus^pos^ cells that display stable/persistent peptide:MHC complexes will be unable to form productive contacts with T cells due to their positioning within the tissue. Previous studies have established that the major population of migratory cells from the infected lung belongs to the CD45^+^ DC lineage, which likely possess advantages in cell surface density of MHC class II, expression of co-stimulatory molecules and co-localization with T effectors^92^. Efforts to study interactions of Venus^pos^ cells with T cells *in vivo* in lymphoid and mucosal tissues will be the focus of future endeavors using a combination of genetically modified viruses, animals and imaging-based approaches.

There are some issues that remain unresolved from this study. Although we^44, 56, 101, 102^ and others^24, 25, 30, 50, 58, 59^ have used fluorescent reporter proteins as valid surrogates to track cells that have encountered viral antigen *in vivo*, it is possible that such reporter viruses underrepresent the absolute abundance and frequency of total antigen-positive cells. First, the signal from Venus may be lost in cells due to quenching or degradation. Second, some phagocytic cells may acquire viral antigen(s) independently of NS1-Venus. For example, membrane fragments released from dying or dead cells will contain HA, neuraminidase and the Matrix-2 ion channel, which may be taken up by lectin-based receptors expressed by some cell types^23, 28, 29^ and may be deficient in uptake of proteins that are not membrane-associated such as NP, polymerase proteins and NS1.

In summary, through the combination of influenza reverse genetics, *in vivo* infection, multi-parameter flow cytometry and antigen-specific CD4 T cells, we have been able to identify many cell types within the respiratory tract that access influenza antigens. Because a subset of these cells display viral peptide:MHC class II complexes and can stimulate influenza-reactive CD4 T cells, they have the potential to interact with previously activated, resident or newly primed CD4 T cells that migrate to the lung after expansion and differentiation. Furthermore, it has been documented that non-neutralizing, influenza-specific antibodies impact protective immunity and disease pathogenesis^103–109^. It will be of interest to ascertain the precise mechanism(s) by which these antibodies influence the abundance, diversity and fate of antigen-bearing cells described here.

## Materials and Methods

### Generation of the NS rescue plasmid containing NS1-Venus fusion

A recombinant A/California/04/09 (H1N1) non-structural (NS) segment, where the C-terminus of NS1 was fused to the Venus fluorescent protein, was engineered as previously described^56, 101^. The recombinant NS segment was synthesized *de novo* (Biomatik) with the appropriate restriction sites for subcloning into the ambisense plasmid- pDZ^110^ to generate the plasmid pDZ-NS-AgeI/NheI. The recombinant NS segment contained the NS1 open reading frame (ORF) without stop codons or splice acceptor sites, followed by AgeI and NheI restriction sites, the porcine teschovirus-1 (PTV-1) 2 A autoproteolytic cleavage site (ATNFSLLKQAGDVEENPGP) and the entire ORF of the nuclear export protein, NEP^56, 101^. The Venus ORF was cloned using the AgeI and NheI sites, into pDZ-NS-AgeI/NheI to generate the pDZ-NS-Venus for virus rescue. Plasmid constructs were confirmed by DNA sequencing (Genewiz).

### Rescue of Venus expressing, pandemic 2009 H1N1 virus and viral kinetics

Recombinant wild-type (WT) and Venus viruses with the genetic backbone of A/California/4_NYICE_E3/09 (H1N1)^111, 112^ were rescued using previously described ambisense pDZ plasmid reverse genetics methods^41, 42, 67, 110^. The viruses were plaque purified and amplified on Madin Darby Canine Kidney (MDCK) cells to generate the viral stocks used in this work. Multicycle virus growth kinetics were performed in MDCK cells (12-well plate format, 5 × 10^5^ cells, triplicates) infected with the indicated viruses at a low multiplicity of infection (MOI) of 0.001 as previously described^56, 113, 114^. At the indicated hours post-infection (hpi), tissue culture supernatants were collected and viral titers were determined by immunofocus assay (fluorescent focus-forming units, FFU/ml) using the mouse monoclonal antibody (MAb), anti-NP (HB-65) as previously described^112, 115, 116^. Mean value and standard deviation (SD) were calculated using Microsoft Excel software.

### Western blot assays

Confluent cultures of MDCK cells were mock infected or infected (MOI 3) with pH1N1-WT or pH1N1-Venus. 24 hpi, cells were lysed in RIPA buffer (25 mM Tris HCl pH 7.6, 150 mM NaCl, 1% NP-40, 1% sodium deoxycholate, 0.1% SDS) and proteins were separated using 10% SDS-polyacrylamide gels and transferred to nitrocellulose membranes. Western blot analysis was performed as previously described^56, 101, 102^, using specific polyclonal antibodies (PAb) for NS1 (PAb; 1–73)^117^, NP^117^, or GFP (PAb; Santa Cruz, sc-8334). A MAb against actin (MAb; Sigma, A1978) was used as an internal loading control. Bound primary antibodies were detected with horseradish peroxidase (HRP)-conjugated antibodies against immunoglobulins of different species (mouse or rabbit), and chemiluminescence using the SuperSignal West Femto maximum-sensitivity substrate (Thermo Scientific) by following the manufacturer’s recommendations.

### Fluorescence and indirect immunofluorescence assay

MDCK cells were mock infected or infected (MOI 3) with pH1N1-WT or pH1N1-Venus. 18 hpi, cells were fixed with 4.0% paraformaldehyde (PFA) and the indirect immunofluorescence analysis was performed as previously described^56, 101, 102^ using primary MAb- anti-NP (HB-65) and Texas Red-conjugated anti-mouse secondary antibody (Dako). The cell nuclei were stained with 4′,6-Diamidino-2-Phenylindole (DAPI, Research Organics). Images were captured using a fluorescence microscope (Nikon Eclipse TE2000) at 10x magnification.

### Plaque assays and viral titrations

Confluent MDCK cell monolayers (6-well plate format, 10^6^ cells/well) were infected with 10-fold serial dilutions of pH1N1-WT or pH1N1-Venus viruses. After infection, monolayers were overlaid with agar and incubated for 3 days at 33 °C, 5% CO_2_. Cells were then fixed with 4% PFA, and the overlays were carefully removed. For visualization of Venus, Phosphate Buffered Saline (PBS) was added and plates were imaged with a Kodak image station (4000 MM Pro molecular imaging system; Carestream Health Inc., NY) and Kodak molecular imaging software (v.5.0.1.30). Fixed cells were then permeabilized (0.5% Triton X-100 in PBS for 15 minutes (min) at room temperature) and prepared for immunostaining as previously described^56, 101, 102, 113^ using the anti-NP MAb (HB-65) and vector kits (Vectastain ABC kit and DAB HRP Subtrate Kit: Vector), according to manufacturer’s specifications.

### Animals and infections

Female C57BL/6 (I-A^b^) were obtained from Charles River Laboratories (Frederick, Maryland) and maintained in a specific-pathogen-free facility at the University of Rochester Medical Center, according to institutional guidelines. Mice were anaesthetized with avertin (2,2,2-Tribromoethanol) via intraperitoneal injection and infected intranasally with 10^5^–10^6^ plaque-forming units (PFU) of pH1N1-Venus. All experimental groups were age matched and used between 10–16 weeks of age.

### Ethics Statement

All mice were maintained in a specific-pathogen free facility at the University of Rochester Medical Center according to institutional guidelines. All animal protocols used in this study adhere to the AAALAC International, the Animal Welfare Act and the PHS Guide, and were approved by the University of Rochester Committee on Animal Resources, Animal Welfare Assurance Number A3291-01. The protocol under which these studies were conducted was originally approved March 4, 2006 (protocol # 2006-030) and has been reviewed and re-approved every 36 months with the most recent review and approval February 6, 2015.

### Cell isolation and staining for flow cytometry

Mice were euthanized at the indicated times post-infection and respiratory and lymphoid tissues were excised. Tissues were minced, followed by enzymatic digestion, where cells from respiratory tissues were digested with a cocktail containing collagenase type II and DNAse I and secondary lymphoid organs were digested with a cocktail containing collagenase type IV and DNase I for 30 min in cell culture media (RPMI 1640 supplemented with L-glutamine, 2.5% FBS, 10 mM HEPES, 1 mg/ml collagenase (Type II or IV) and 30 μg/ml DNase I) at 37 °C. Digested tissues were then crushed, passed through a 40 μm nylon mesh filter and rinsed using nutrient-supplemented Dulbecco’s modified Eagle medium (DMEM) (Gibco), including 1% gentamycin and 10% heat-inactivated fetal bovine serum (FBS). Single cell suspensions were then depleted of red blood cells by treatment with ACK lysis buffer (0.15 M NH_4_Cl, 1.0 mM KHCO_3_, 0.1 mM Na_2_-EDTA in H_2_O, pH 7.2) when necessary. For analytical flow cytometry experiments, cells were seeded in a 96 well, v-bottom plate at a concentration of 10^6^ cells per well and washed twice in PBS, followed by a 30 min incubation at 4 °C with fixable live/dead Aqua (Life Technologies) to exclude non-viable cells according to manufacturer instructions. Cells were then washed in stain buffer (PBS supplemented with 2% heat-inactivated FBS and 0.01% NaN_3_) followed by 15 min incubation at 4 °C with purified rat anti-mouse CD16/CD32 (mouse BD Fc Block, clone 2.4G2) to reduce nonspecific binding of antibodies. Cells were stained for 30 min at 4 °C in the dark using antibodies targeting the following markers: CD11b (M1/70, BD Pharmingen, APC/Cy7), CD103 (M290, BD Horizon, BV786), CD45 (30-F11, Tonbo, V450), CD11c (HL3, BD Pharmingen, PE/Cy7), Siglec F (E50-2440, BD Horizon, PE-CF594), I-A^b^ (M5/114.15.2, BD Horizon, BV711), Ly6C (AL-21, BD Horizon, BV605), Ly6G (1A8, Biolegend, BV650), CD64 (X54-5/7.1, PE) and HA (SC70-5B03, P. Wilson Lab (U. Chicago), conjugated to Alexa Fluor 647)^75^. Data was acquired using a BD LSR-II instrument, configured with 488 (blue), 633 (red), 407 (violet), and 532 (green)-nm lasers. Data was analyzed using Flowjo software (Tree Star Inc.), version 10. For Fluorescence-Activated Cell Sorting (FACS) experiments, a BD FACSAria instrument with 488-, 633-, 407-, and 522-nm lasers was used, and events collected through a 100 μm nozzle under purity settings.

### Enzyme-Linked ImmunoSpot (ELISpot) assays to determine antigen presentation by Venus^pos^ cells following infection

To enumerate potential Venus^pos^ APC displaying peptide: MHC class II complexes, influenza-specific CD4 T cells were generated as follows: Age and sex-matched C57BL/6 mice were primed by subcutaneous injection of the rear footpad containing an emulsion of the following three immunodominant, I-A^b^-restricted influenza peptides (5 nmol each) in Addavax (50% v/v) containing 25 μg CpG ODN 1826 (InvivoGen): NP_261-278_ (RSALILRGSVAHKSCLPA), NP_413-436_ (SVQRNLPFERATVMAAFSGNNEGR) and NA_172-192_ (SRFESVAWSASACHDGINWLT). 10–13 days post-immunization, cells were isolated from the draining popliteal lymph nodes. In some experiments, as indicated in the Figure Legends, antigen-experienced CD4 T cells were isolated using negative paramagnetic bead selection (STEMCELL Technologies Inc, Vancouver, Canada) and then used directly in the ELISpot assay. Alternatively, total CD4 T cells were purified using negative paramagnetic bead selection (Miltenyi Biotec, CA, USA) and then expanded by culture *in vitro* for 12 days with naïve syngeneic donor APCs (4:1 ratio of splenic APC to CD4 cells) in the presence of peptides (2 μM each of NP_261-278_, NP_413-436_ and NA_172-192_) and recombinant IL-2 protein (10 U/ml), washed and then plated into the ELISpot assay.

To isolate Venus^pos^ APC, a separate cohort of mice were infected (10^5^ PFU) using pH1N1-Venus. 2 dpi, lung tissues were harvested, pooled and processed into single cell suspensions as previously described. Cell suspensions were stained with fixable live/dead Aqua, washed (1X DPBS, supplemented with 2% HI-FBS) and blocked with Fc Block prior to staining using antibodies targeting CD45 and HA as previously described. Alternatively, cDC and MΦ populations (aMΦ and iMΦ) were separately purified using CD11c bead enrichment using positive selection (Miltenyi Biotec, CA, USA) and stained using the following antibodies: CD45, CD24 (M1/69, BD Pharmingen, Alexa Fluor 700), CD64, CD11b, CD103, Siglec F). Cells were resuspended in Hank’s Balanced Salt Solution containing 10% HI-FBS, sorted to high purity and plated in graded numbers, in duplicate as a co-culture with a lawn of influenza-specific CD4 T cells (55,000–75,000 per well) for 20 hrs at 37 °C, 5% CO_2_ in an ELISpot assay, as previously described^83, 118^. Quantification of IFN-γ-secreting cells was performed with an Immunospot reader series 2A, using Immunospot software, version 5.0.9.19. Based on our previous studies, we estimate that on average, this assay has a 10% efficiency in eliciting cytokine production in a co-culture of cells with APC in the continual presence of exogenous, antigenic peptide in the overnight culture.

### Data Availability

All data generated or analyzed during this study are included in this published article (and its Supplementary Information files).

## Electronic supplementary material

Supplementary Info
