# Supplementary material for: Pandemic 2009 H1N1 Influenza Venus reporter virus reveals broad diversity of MHC class II-positive antigen-bearing cells following infection in vivo
Source: Sci Rep. 2017 Sep 7;7:10857. doi: 10.1038/s41598-017-11313-x (PMC5589842; doi:10.1038/s41598-017-11313-x)

SUPPLEMENTARY FILES

Pandemic 2009 H1N1 Influenza Venus reporter virus reveals broad diversity of MHC class II-positive antigen-bearing cells following infection in vivo

Anthony DiPiazza[1], Aitor Nogales[1], Nicholas Poulton[1], Patrick C. Wilson[2], Luis Martínez-Sobrido[1], Andrea J. Sant[1*]

[1]Department of Microbiology and Immunology, David H. Smith Center for Vaccine Biology and Immunology, University of Rochester Medical Center, Rochester, NY, USA [2]Department of Medicine, Section of Rheumatology, The Committee on Immunology, The Knapp Center for Lupus and Immunology Research, The University of Chicago, IL 60637

Supplemental Figure 1. Representative gating schematic for identification of Venus^{pos} cells. Cell types in (**A**) lung and (**B**) lung draining lymph node (mediastinal lymph node) were resolved using flow cytometry as described in Figure 4 and materials and methods. Venus gates were drawn based on WT-matched (lung) or mock-infected controls (mLN) to account for possible autofluorescence as a result of the infection. Dot plots are representative of all experiments.

Supplemental Figure 2. Lung cell composition comparison elicited by WT and pH1N1-Venus infection. Age and sex-matched C57BL/6 (I-A^b) mice were infected with 10^6 PFU WT or Venus-encoding pH1N1 virus. 2 dpi, lungs were processed and prepared for flow cytometry. Data is representative of pooled responses from 4 animals per group.

Supplemental Figure 3. Composition of Venus^{pos} cells in the lung early following infection using a tenfold reduction of virus. (**A**) Direct comparison of the Venus^{pos} cell composition 2 dpi from lung tissue using either 10^6 or 10^5 PFU pH1N1-Venus per animal. Pie charts represent a composite from either multiple independent experiments where lung tissues were pooled prior to staining and analysis (10^6 PFU) or were averaged from multiple, individual samples (10^5 PFU) from the same experiment. (**B**) Composition of Venus^{pos} cells from individual animals infected with 10^5 PFU pH1N1-Venus. Pie charts from individual animals (n = 4) highlight the remarkable reproducibility using a tenfold reduction of infectious virus.

Supplemental Figure 4. Representative gating schematic for identification of infected cells. Cell types were resolved using flow cytometry as described in Figure 4 and materials and methods. Venus gates were drawn based on WT-matched controls to account for possible autofluorescence as a result of the infection. Two-dimensional (Venus versus HA) dot plots with quadrant gating illustrates how Venus^{pos}/HA^{neg} and Venus^{pos}/HA^{pos} cells were identified. HA fluorescence minus one (FMO) control demonstrates proper color compensation and all positive events within the HA^{pos} gate are presumably infected.

Supplemental Figure 5. FACS purified Venus^{pos}HA^{neg} cells are not kinetically delayed in surface HA expression. Sorted cells were rested in primary culture media and incubated at 37°C, 5% CO_2 for 16-18 h. Cells were then counterstained with an anti-HA mAb (SC70 5B03) and evaluated for surface HA expression. Data is representative from two independent experiments using cells pooled from 3-4 animals, with each dataset overlayed on the same dot plot and depicted in blue or red.

SUPPLEMENTAL FIGURE 1

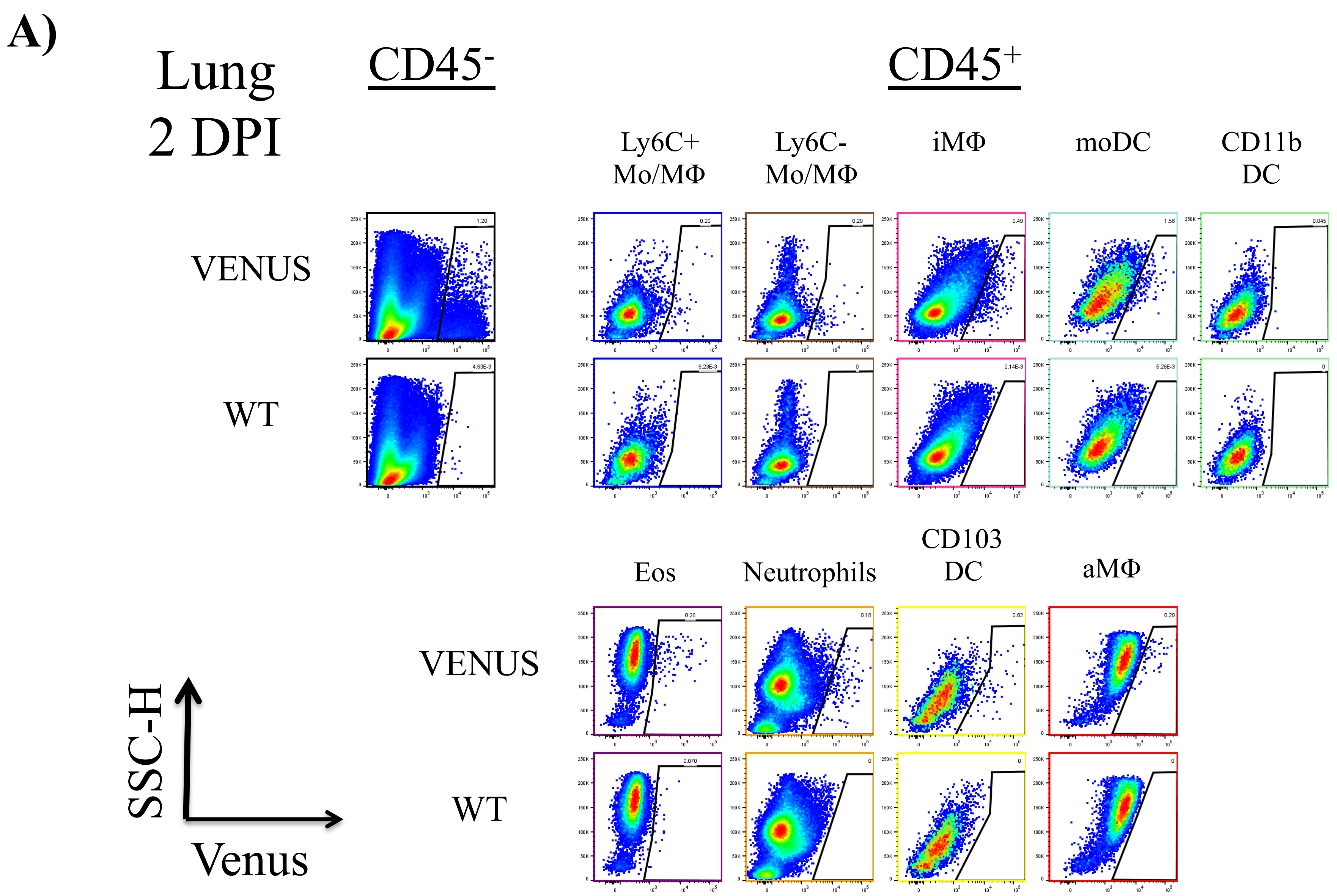

SUPPLEMENTAL FIGURE 1

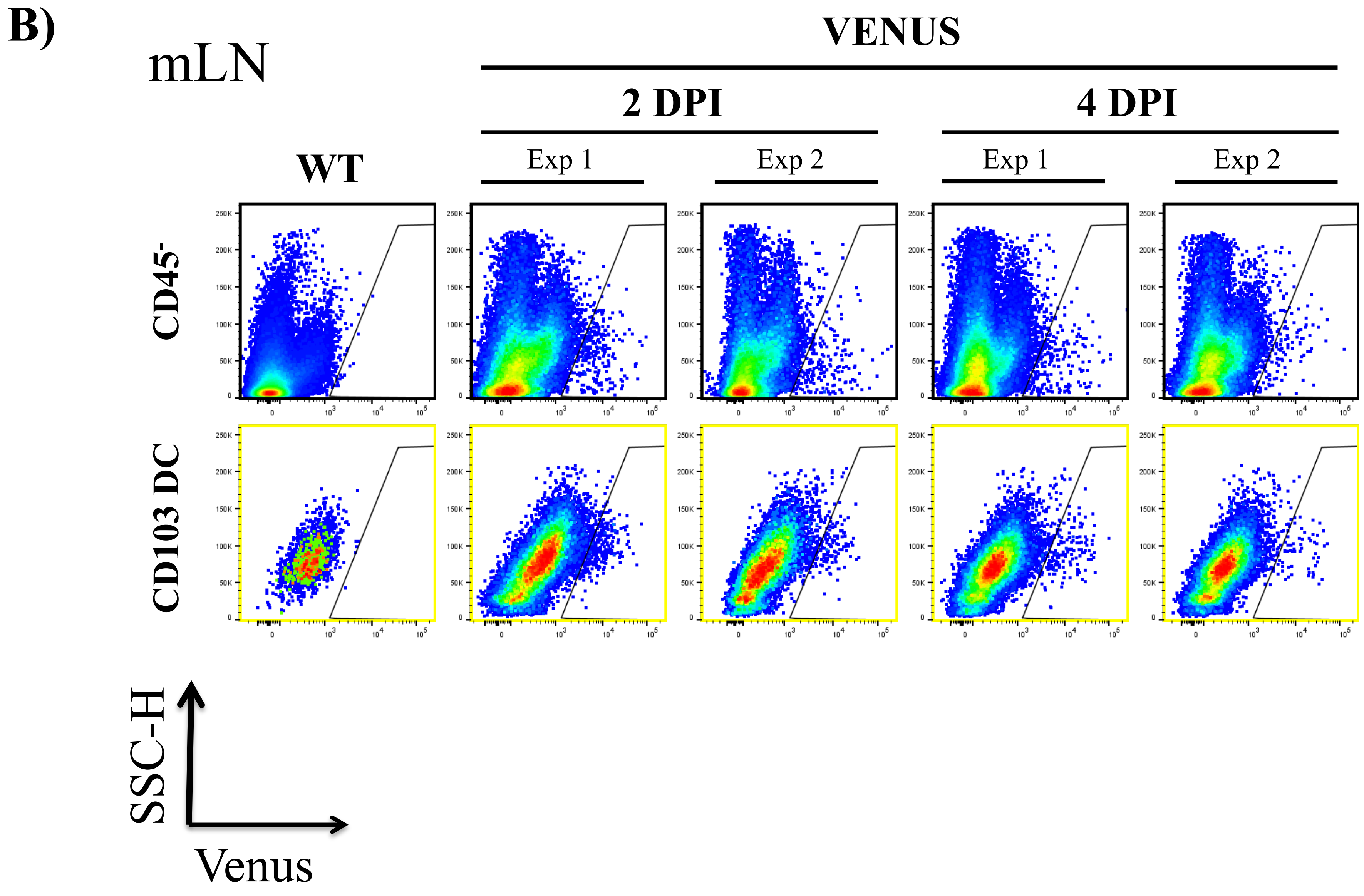

SUPPLEMENTAL FIGURE 2

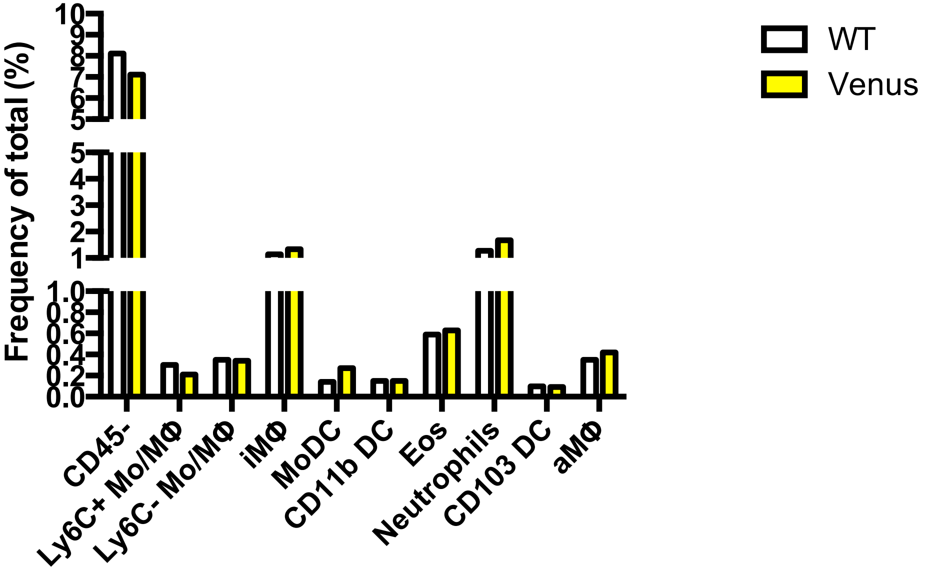

SUPPLEMENTAL FIGURE 3

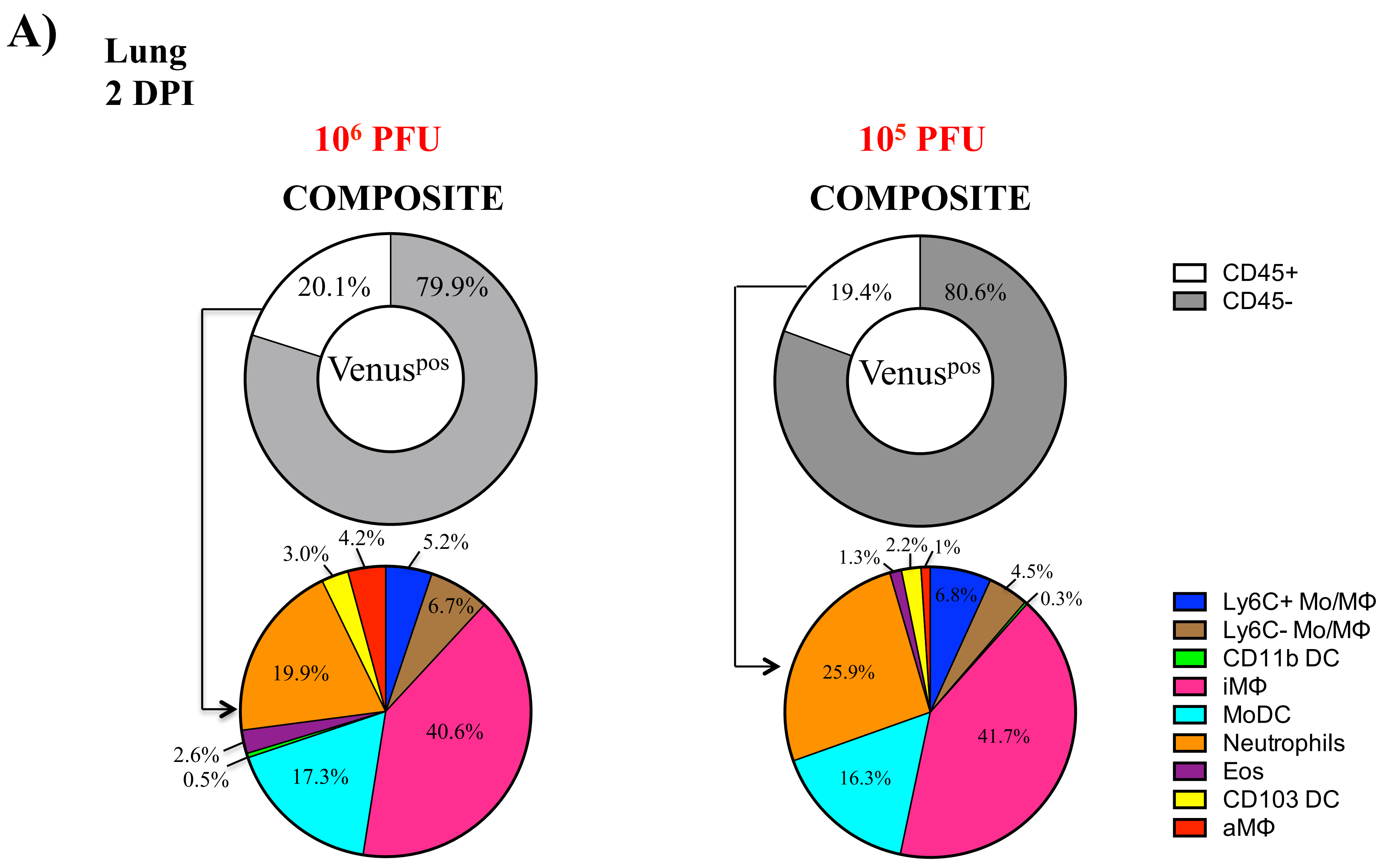

SUPPLEMENTAL FIGURE 3

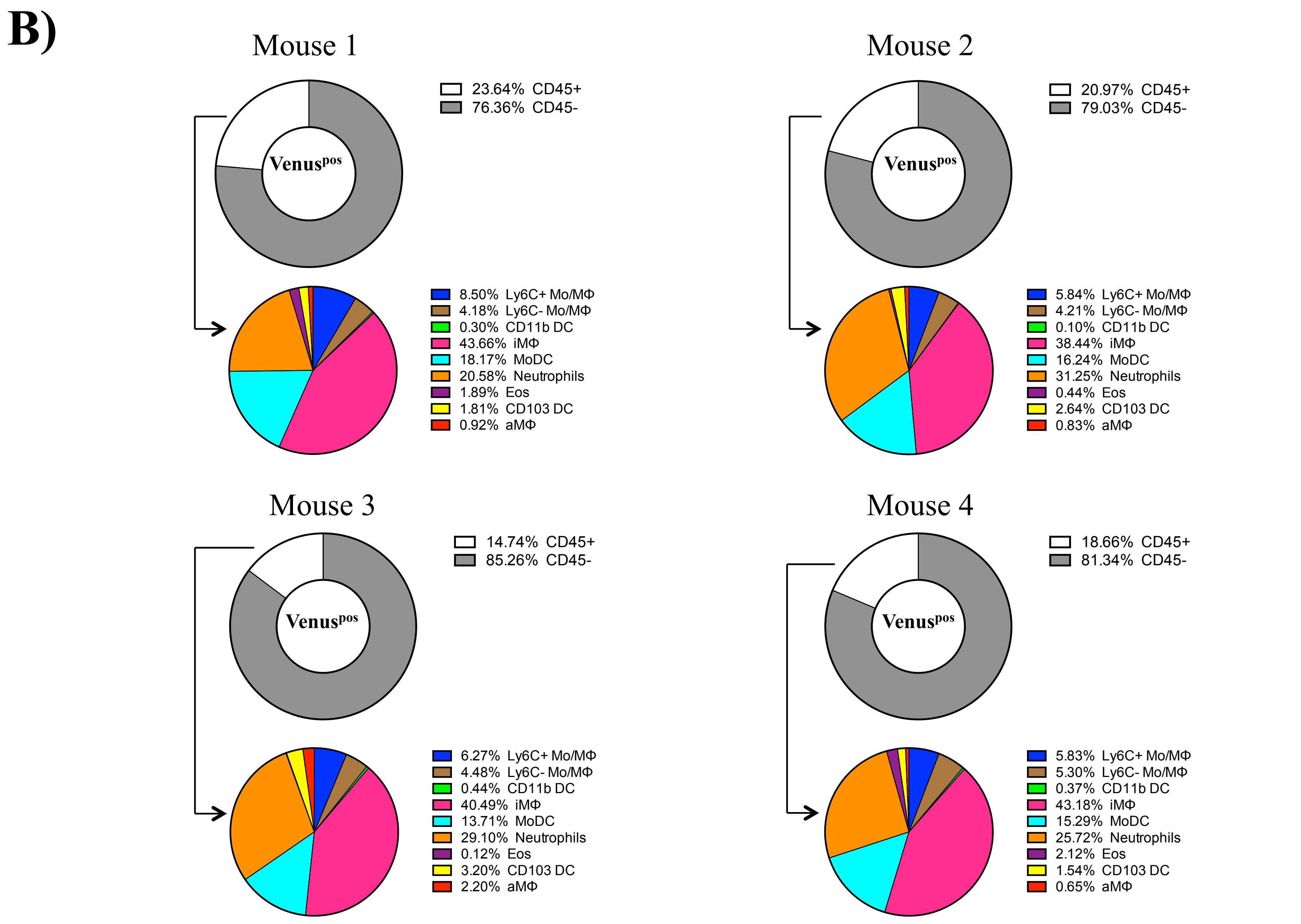

SUPPLEMENTAL FIGURE 4

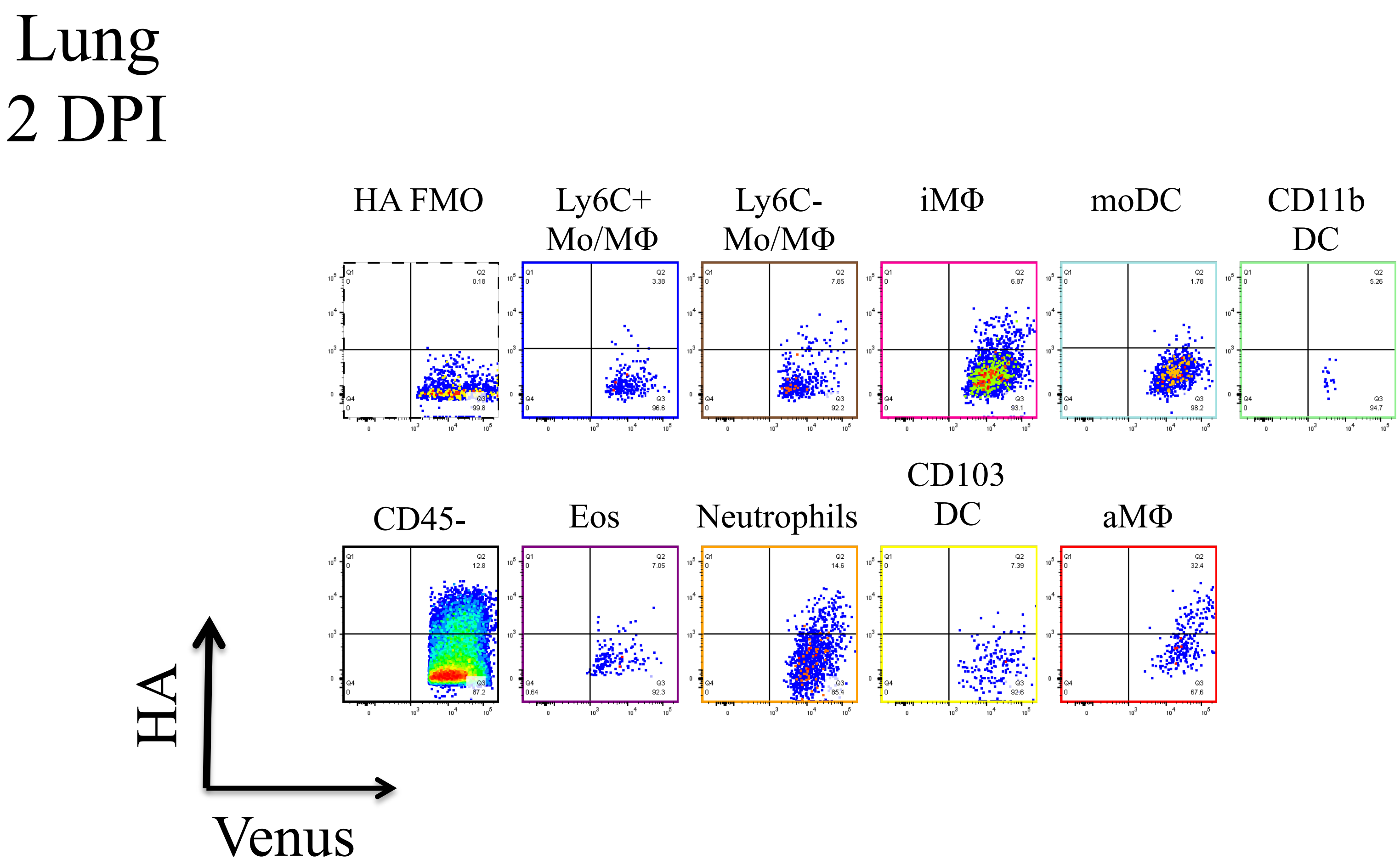

SUPPLEMENTAL FIGURE 5

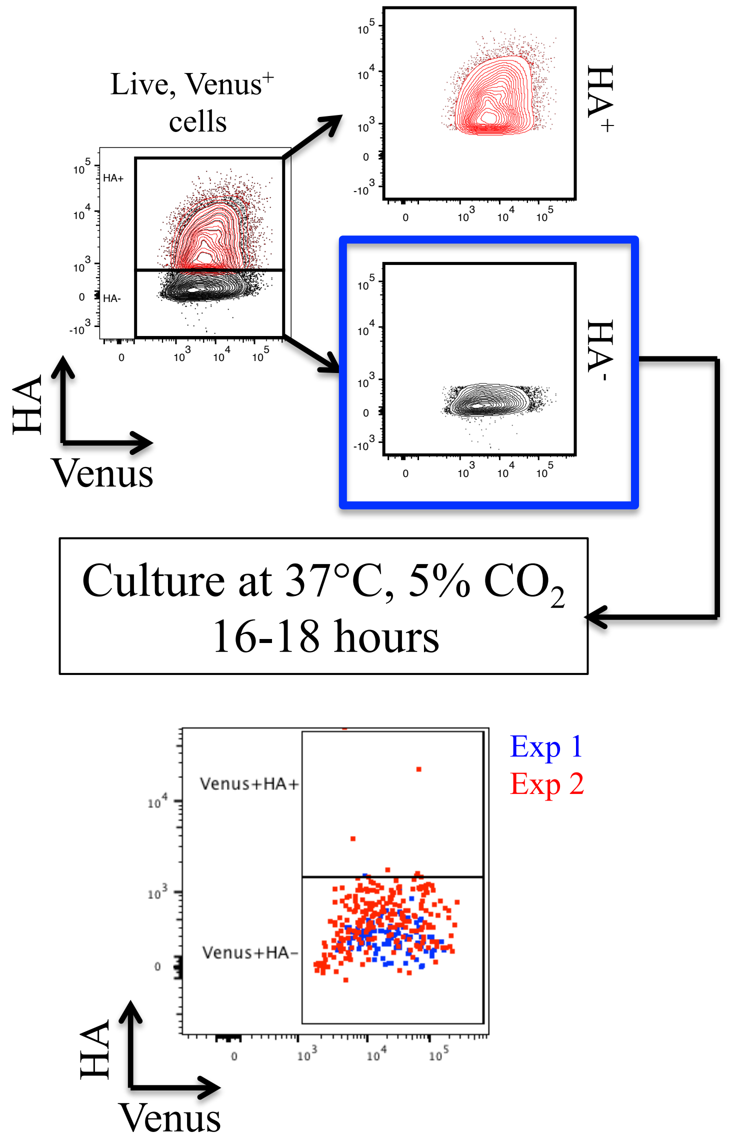

Supplement: Supplementary file 1 — Supplementary Info [file 41598_2017_11313_MOESM1_ESM.pdf]
